# Analytical Frameworks and Outcome Measures in Economic Evaluations of Digital Health Interventions: A Methodological Systematic Review

**DOI:** 10.1177/0272989X221132741

**Published:** 2022-10-19

**Authors:** Valerio Benedetto, Luís Filipe, Catherine Harris, Joseph Spencer, Carmel Hickson, Andrew Clegg

**Affiliations:** Synthesis, Economic Evaluation and Decision Science (SEEDS) Group, Health Technology Assessment (HTA) Unit, Applied Health Research hub, University of Central Lancashire, Preston, Lancashire, UK; Methodological Innovation, Development, Adaptation and Support (MIDAS) Theme, National Institute for Health and Care Research Applied Research Collaboration North West Coast (NIHR ARC NWC), Liverpool, Merseyside, UK; Methodological Innovation, Development, Adaptation and Support (MIDAS) Theme, National Institute for Health and Care Research Applied Research Collaboration North West Coast (NIHR ARC NWC), Liverpool, Merseyside, UK; Department of Health Research, Faculty of Health & Medicine, Lancaster University, Lancaster, Lancashire, UK; Synthesis, Economic Evaluation and Decision Science (SEEDS) Group, Health Technology Assessment (HTA) Unit, Applied Health Research hub, University of Central Lancashire, Preston, Lancashire, UK; Methodological Innovation, Development, Adaptation and Support (MIDAS) Theme, National Institute for Health and Care Research Applied Research Collaboration North West Coast (NIHR ARC NWC), Liverpool, Merseyside, UK; Methodological Innovation, Development, Adaptation and Support (MIDAS) Theme, National Institute for Health and Care Research Applied Research Collaboration North West Coast (NIHR ARC NWC), Liverpool, Merseyside, UK; Research Facilitation and Delivery Unit (RFDU), Applied Health Research hub, University of Central Lancashire, Preston, Lancashire, UK; Public Advisers’ Forum, National Institute for Health and Care Research Applied Research Collaboration North West Coast (NIHR ARC NWC), Liverpool, Merseyside, UK; Synthesis, Economic Evaluation and Decision Science (SEEDS) Group, Health Technology Assessment (HTA) Unit, Applied Health Research hub, University of Central Lancashire, Preston, Lancashire, UK; Methodological Innovation, Development, Adaptation and Support (MIDAS) Theme, National Institute for Health and Care Research Applied Research Collaboration North West Coast (NIHR ARC NWC), Liverpool, Merseyside, UK

**Keywords:** cost-effectiveness analysis, digital health, economic evaluations, outcome measures, QALY

## Abstract

**Background:**

Digital health interventions (DHIs) can improve the provision of health care services. To fully account for their effects in economic evaluations, traditional methods based on measuring health-related quality of life may not be appropriate, as nonhealth and process outcomes are likely to be relevant too.

**Purpose:**

This systematic review identifies, assesses, and synthesizes the arguments on the analytical frameworks and outcome measures used in the economic evaluations of DHIs. The results informed recommendations for future economic evaluations.

**Data Sources:**

We ran searches on multiple databases, complemented by gray literature and backward and forward citation searches.

**Study Selection:**

We included records containing theoretical and empirical arguments associated with the use of analytical frameworks and outcome measures for economic evaluations of DHIs. Following title/abstract and full-text screening, our final analysis included 15 studies.

**Data Extraction:**

The arguments we extracted related to analytical frameworks (14 studies), generic outcome measures (5 studies), techniques used to elicit utility values (3 studies), and disease-specific outcome measures and instruments to collect health states data (both from 2 studies).

**Data Synthesis:**

Rather than assessing the quality of the studies, we critically assessed and synthesized the extracted arguments. Building on this synthesis, we developed a 3-stage set of recommendations in which we encourage the use of impact matrices and analyses of equity impacts to integrate traditional economic evaluation methods.

**Limitations:**

Our review and recommendations explored but not fully covered other potentially important aspects of economic evaluations that were outside our scope.

**Conclusions:**

This is the first systematic review that summarizes the arguments on how the effects of DHIs could be measured in economic evaluations. Our recommendations will help design future economic evaluations.

**Highlights:**

- Using traditional outcome measures based on health-related quality of life (such as the quality-adjusted life-year) may not be appropriate in economic evaluations of digital health interventions, which are likely to trigger nonhealth and process outcomes.
- This is the first systematic review to investigate how the effects of digital health interventions could be measured in economic evaluations.
- We extracted and synthesized different arguments from the literature, outlining advantages and disadvantages associated with different methods used to measure the effects of digital health interventions.
- We propose a methodological set of recommendations in which 1) we suggest that researchers consider the use of impact matrices and cost-consequence analysis, 2) we discuss the suitability of analytical frameworks and outcome measures available in economic evaluations, and 3) we highlight the need for analyses of equity impacts.

The role of technology in health care is ever growing. Technological innovations have introduced new treatments and diagnostic tests that affect people’s quality of life and life expectancy. They are also changing how health care services are used, allowing individuals to be empowered in monitoring and managing their own care.^1–3^

*Digital health* is a wide-encompassing term that includes multiple and diverse interventions based on information and communications technologies, spanning over mobile health (or mHealth), telemedicine, and telehealth.^4^ Reducing transportation costs, inefficiencies, hospital stays, and time to diagnosis are some of the potential gains attributable to digital health interventions (DHIs).^5–7^ DHIs can widen the accessibility to health care services, extending their reach to remote areas or, as in the COVID-19 pandemic, to people in self-isolation. However, these benefits come at a price, for example, the costs of the new technologies or adapting to new processes.^8^ Quality of care may decrease if the new DHIs are not a perfect substitute for the existing alternative or if users and health care professionals struggle to fully adapt to the new procedures.^8^ These drawbacks potentially affect the safety, acceptability, and effectiveness of the new technologies. Inequality and ethical issues may also arise, as individuals are likely to differ in the way they access and accept the use of a digital health technology.^8–10^

As with any new intervention, the natural tradeoffs in DHIs call for economic evaluations estimating their costs and consequences.^9^ The effects triggered by DHIs on accessibility, acceptability, quality, and costs^8,11^ increase the number of key outcomes to consider. Process outcomes are likely to emerge^6^ (e.g., number of face-to-face visits) and outweigh the value of health-related quality of life (HRQoL) outcomes, which sometimes share only a tenuous link with DHIs.^12^ Consequently, the ability of standard outcome measures based on HRQoL, such as the quality-adjusted life year (QALY), to capture all of the relevant outcomes of DHIs is a matter of debate.^13^

While the simplicity of the QALY contributes to its wide acceptance and key role in health care decision making,^14^ limitations have emerged. These have ranged from theoretical issues (such as the lack of correspondence between QALY-underlying expected utility and actual individuals’ behavior^14–18^) to more methodological aspects (e.g., diverging utility values obtained from adopting different eliciting techniques^15–17,19,20^). Equity concerns have also been voiced, particularly when interventions not likely to substantially improve life expectancy nor health conditions (and thus yielding lower QALYs) may still be important for specific populations.^15–17,21^

Specific problems arise in the context of DHIs too. DHIs are multidimensional in the way they produce multiple effects to numerous stakeholders. The most common examples pertain to the user’s perspective, ranging from more tangible effects, such as those related to reduced waiting or travel time, faster diagnosis, and better access to health care services,^5–7,12^ to less tangible ones, like the sense of reassurance or anxiety triggered by the flow of information on personal health.^6,22^ The perspectives of health care professionals and managers can also be taken into account (e.g., How do they accept or are they willing to use a DHI? Which educational effects can be reaped?), as well as the perspective of the whole health care system (e.g., how can the implementation of a DHI be scaled up?).^13,23^ Further perspectives that go beyond the interaction between users and health care professionals may be considered relevant, such as those of caregivers or other users.^22^

In addition, DHIs can be applied to multiple health areas. This affects the generalizability of their evaluations, which may also fail to capture the long-term and evolving effects of DHIs.^5,22,24^ The demand of health care services may also change over time, as the use of DHIs can uncover needs that traditional interventions are not able to meet.^23^

These challenges indicate that one-size-fits-all rules for economic evaluations of DHIs may not be sensible. In the complexity of interactions created by DHIs,^25,26^ HRQoL-informed QALYs and other generic outcome measures may not fully capture externalities (e.g., effects on caregivers), nonhealth factors (e.g., travel time), network effects (e.g., as the number of users increases, the overall digital health technology improves), and other process outcomes.^5,6,22,27^

Economic evaluations of DHIs and systematic reviews assessing their quality and findings^28,29^ have proliferated, while suggestions addressing methodological challenges are emerging.^30^ However, to our knowledge, no review has synthesized arguments on how the effects of DHIs could be measured in economic evaluations, including whether HRQoL-informed QALYs and other generic outcome measures could be valid metrics in this field. In this review, we intend to address this gap by collecting, assessing, and synthesizing arguments on how to measure the effects of DHIs in economic evaluations, as we focus on the arguments on the choice and use of analytical frameworks and outcome measures. Then, we use the findings to create a set of methodological recommendations that can guide future economic evaluations of DHIs.

## Methods

The systematic review process followed a predetermined protocol (registered on PROSPERO as CRD42021243636) and standard reporting guidance^31^ (Supplementary Table S1).

### Search Strategy

We searched 5 electronic databases, specifically MEDLINE (Ovid), Embase (Ovid), Cochrane Database of Systematic Reviews and Cochrane Central Register of Controlled Trials (Cochrane Library), International Health Technology Assessment Database, and the NHS Economic Evaluation Database. Search terms used included “digital health” and common alternatives terms (e.g., telemedicine, eHealth, telehealth, mHealth), along with “economic”, “quality-adjusted life year,”“value,” and “outcome”. The search strategies used are presented in Supplementary Tables S2 to S6. The searches were run on February 22, 2021, and no date limits were applied.

Gray literature searches were conducted on health economic websites, including International Society for Pharmacoeconomics and Outcomes Research (ISPOR), international Health Economics Association (iHEA), and the Office of Health Economics (OHE). The websites were searched via the Google search engine, due to limitations in search functionality on the websites themselves. These searches were run by 2 of the coauthors on March 26 (L.F.) and March 31, 2021 (V.B.), using key synonyms for “digital health” (Supplementary Table S7).

Backward citation searches were also conducted by checking the references of the studies included in the analysis following the initial searches and screening. References citing the studies included in the analysis were identified by running forward citation searches in Scopus, Web of Science, and Google Scholar on June 17, 2021.

### Study Selection

The main criterion for study inclusion was the presence of a discussion of theoretical and empirical challenges of, and/or the advantages and disadvantages associated with, the measurement, valuation, and use of outcome measures, including the choice of analytical frameworks, for economic evaluations of DHIs. This represented our outcome in an adapted version of the population (general population), intervention (any DHIs), comparator (any), and outcome model (PICO). We considered any empirical and nonempirical studies (e.g., systematic reviews, economic evaluations, theoretical and methodological studies), except abstracts. Only records in English were included.

Those records retrieved by the multidatabase searches were de-duplicated and then screened. To determine eligibility, 4 coauthors (V.B., L.F., C.Ha., J.S.) used a prepiloted screening tool (Supplementary Table S8) as part of a 2-stage screening process managed in EndNote:

1. Records were split in 4 batches, with the title and abstract of each record screened by 1 coauthor, and a random sample (20% of the batch size) cross-screened by another coauthor.
2. The full text of selected records was then screened independently by 2 coauthors.

### Data Extraction

The same 4 coauthors extracted data from the selected studies and validated each other’s extractions using a prepiloted Excel template that focused on the following:

• Aim and design
• Arguments on measurement, valuation, and use of outcome measures, including:
 ○ instruments to collect health states data,
 ○ techniques used to elicit utility values or weights, and
 ○ generic and disease-specific outcome measures
• Other arguments on outcome measures (e.g., analysis and interpretation of results) or analytical frameworks

This list was updated during the data extraction process as new relevant items were identified. Any discrepancy in the study selection was resolved through discussions, with oversight by another coauthor (A.C.). The protocol and this article were reviewed by a public adviser (C.Hi.), whose involvement is detailed in Supplementary Table S9.

### Quality Assessment

Because our review focused on the arguments presented in the studies, a traditional assessment of the overall study quality was out of scope. Traditional checklists that focus on the quality of the studies’ design and methodology may not be appropriate to review theoretical or qualitative evidence.^32,33^ Therefore, the arguments were qualitatively assessed in our data synthesis.

### Data Synthesis

We undertook a narrative synthesis of the arguments presented in the included studies by relevant methodological areas. This synthesis informed the development of a 3-stage set of recommendations that can help design future economic evaluations of DHIs.

### Role of Funding Source

The funder source had no role in study design, data collection and analysis, decision to publish, or preparation of the article.

## Results

### Search Results

We identified 15,050 results, of which 3,641 were duplicates. Thirty-nine records were selected for full-text screening. Further records were screened through backward (*n* = 16) and forward (*n* = 718) citation searching and gray literature searching (*n* = 212). From those, an additional 19 records were selected for full-text screening (thus 58 in total).

**Figure 1:**
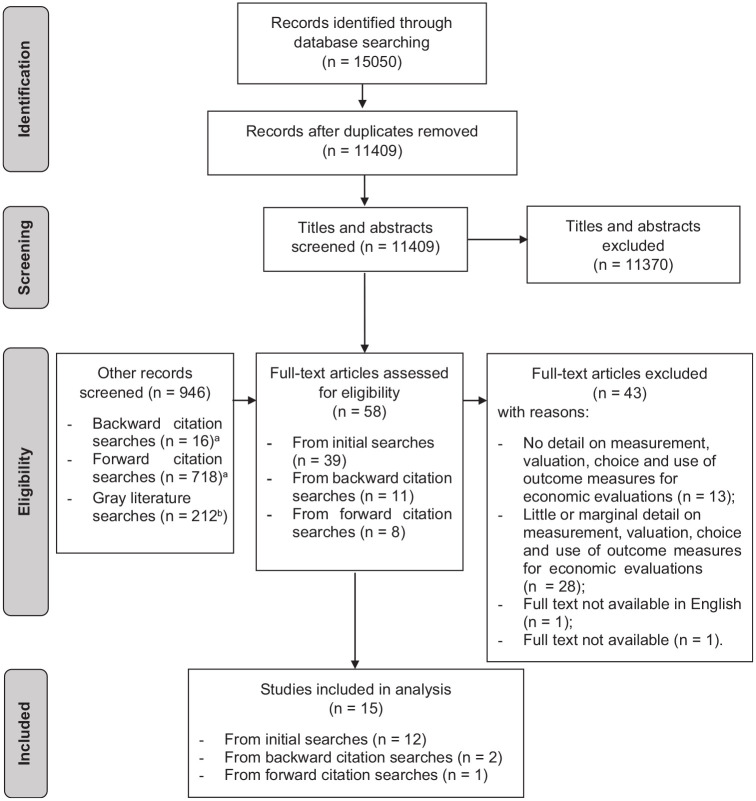
Flowchart reporting search and screening processes identifying included studies.^31^ ^a^Records identified through the backward and forward citation searches were screened first in terms of their title/abstract and, if relevant, also in terms of their full text. ^b^This figure is approximate as the number of results retrieved by the Google search engine tends to vary rapidly.

Following full-text screening, 15 studies were included in the analysis, as summarized in the Preferred Reporting Items for Systematic Reviews and Meta-Analyses (PRISMA) flowchart^31^ (Figure 1). The reasons for the exclusion of the other 43 records are listed in Supplementary Table S10.

### Overall Summary of Included Studies

The included studies were published between 1997 and 2021. All had a theoretical or methodological design: 6 were (or included) reviews of the literature^5,7,13,24,25,34^, 6 were theoretical studies or had a theoretical component,^6,12,22,26,27,35^ 2 proposed theoretical frameworks,^36,37^ and 1 was a methodological guideline.^23^

The arguments extracted from the included studies pertained to analytical frameworks (from 14 studies, equal to 93%), generic outcome measures (5 studies, 33%), techniques used to elicit utility values (3 studies, 20%), and disease-specific outcome measures and instruments to collect health states data (2 studies, 13%). The characteristics of the included studies are summarized in Table 1.

**Table 1:** Summary of Included Studies (*N* = 15)

First Author (Year)	Aim	Design	Were Any of the Extracted Arguments Relevant to . . .				
			Analytical Frameworks?	Instruments to Collect Health States Data?	Techniques Used to Elicit Utility Values?	Generic Outcome Measures?	Disease-Specific Outcome Measures?
Angjellari-Dajci (2013)^36^	To provide a framework for benefit-cost analysis for the economic evaluation of telehealth and face-to-face interventions for people with autism spectrum disorders	Theoretical framework	Yes	No	No	No	No
Bergmo (2014)^25^	To review the use of QALYs in economic evaluations of telehealth	Literature review	Yes	No	Yes	Yes	Yes
Bergmo (2015)^26^	How to apply economic evaluation methods in the eHealth field	Theoretical study	Yes	No	No	Yes	No
Bongiovanni-Delarozière (2017)^34^	To review economic evaluations of telemedicine and define a standardized framework for economic evaluation	Systematic review/theoretical study	Yes	Yes	No	No	No
Davalos (2009)^5^	To review the economic literature and research guidelines in telemedicine	Literature and guidelines review	Yes	No	No	No	Yes
Kolasa (2020)^13^	To describe the characteristics of specific DHIs guidelines and criteria and methods used in the evaluation of DHIs	Systematic review of assessment frameworks	Yes	No	No	Yes	No
LeFevre (2017)^35^	How to select methods for economic evaluation and financial evaluation of DHIs	Guideline/theoretical study	Yes	No	No	No	No
LeGoff-Pronost (2010)^27^	To describe a framework for the economic evaluation of telemedicine networks	Theoretical study with applied case study	Yes	No	No	No	No
McIntosh (1997)^6^	To illustrate the challenges in economic evaluations of telemedicine interventions	Theoretical study	Yes	No	Yes	Yes	No
McNamee (2016)^22^	To focus on the key issues of economic evaluations of DHIs, by describing guides and analytical frameworks for complex interventions and proposing key decision points	Theoretical study	Yes	No	No	No	No
Mistry (2012)^7^	To review economic evaluations of telemedicine interventions and their adherence to reporting guidelines	Systematic review	No	Yes	No	No	No
NICE (2018)^37^	To describe the standard evidence used to demonstrate the value of digital health technologies in the United Kingdom health and social care system	Theoretical framework	Yes	No	No	No	No
Ohinmaa (2001)^23^	To provide an approach for the assessment of telemedicine interventions	Methodological guideline	Yes	No	No	Yes	No
Reardon (2005)^24^	To review literature on economic studies of telemedicine and provide strategies for improvement of future research	Literature review	Yes	No	No	No	No
Snoswell (2017)^12^	To describe the methods of economic evaluation of telehealth interventions	Theoretical study	Yes	No	Yes	No	No
Yes, *n* (%)			14 (93)	2 (13)	3 (20)	5 (33)	2 (13)

DHIs, digital health interventions; NICE, National Institute for Health and Care Excellence; QALY, quality-adjusted life year.

### Synthesis of Arguments

#### Analytical frameworks

Economic evaluations in digital health are challenging, as DHIs can be complex, involve multiple stakeholders,^25,26^ and produce time-changing effects.^22,34^ As their impact on health outcomes may be indirect, using surrogate outcome measures may be necessary, although they may be weakly associated with health outcomes, as underlined by Ohinmaa et al.^23^

While the use of traditional frameworks for economic evaluations is advocated in methodological guidelines, as in the guideline by the National Institute for Health and Care Excellence (NICE) in the United Kingdom,^37^ alternatives exist to deal with the diversity of outcomes and corresponding measurement challenges.^22^

Below, we summarize the arguments we extracted from the included studies, organized by each analytical framework that can be adopted in economic evaluations of DHIs.

##### Cost-consequence analysis (*n* = 3 studies)

The use of cost-consequence analyses (CCAs) is suggested by NICE^37^ when DHIs trigger nonhealth outcomes. According to McIntosh and Cairns,^6^ CCAs can act as a “balance sheet,” which highlights the variety of outcomes attributable to DHIs, identifies data gaps and critical variables for sensitivity analyses, and helps in deciding on the appropriate units of analysis when monetary and nonmonetary outcomes exist. The authors emphasized that in CCAs, the relevance of the tradeoffs between the different costs and consequences is not evident^6^ and relies on the decision makers’ judgment as underlined by Snoswell et al.^12^

##### Cost-benefit analysis (*n* = 7 studies)

The use of monetary metrics, which facilitates cross-area comparisons, is considered an important advantage of cost-benefit analyses (CBAs) by Reardon.^24^ Another advantage considered by this author is the possibility of capturing a broad range of costs and outcomes associated with DHIs.^24^ These can be captured by eliciting the willingness to pay (WTP) of digital health users on factors such as access to health services, ability to measure their own health status, reduced time for appointments, productivity, and efficiency gains.^12,25,35^

However, other studies point to the limits of using CBAs. As shown by Davalos et al.^5^ and Bongiovanni-Delarozière and Le Goff-Pronost,^34^ asking users to supply information about their WTP for different factors and attempting to convert health outcomes into monetary units can be challenging. Unlike other types of tradable commodities, health outcomes are not typically attached to a visible price, which may complicate the valuation of health improvements generated by DHIs, as stressed by Reardon and Angjellari-Dajci et al.^24,36^

##### Cost-effectiveness analysis (*n* = 3 studies)

Reardon^24^ provided insights on the importance of choosing the outcome measure in cost-effectiveness analyses (CEAs). For example, measuring access to care using the number of appointments may overlook how DHIs trigger fewer appointments in the first place.^24^ Another well-known limitation of CEAs pointed out by Reardon,^24^ not confined to digital health, is the lack of cross-area comparability of their findings.

Besides cost-effectiveness, LeFevre et al.^35^ argued that the financial impact and equitable distribution of costs and consequences across the users of DHIs are relevant. According to these authors, extended CEAs can investigate these equity impacts by exploring the role of different health and social determinants across subgroups.^35^ For example, McIntosh and Cairns^6^ emphasized how, in measuring the value of improving access to health care services, a greater weight can be placed on the gains of those living in remote areas.

##### Cost-utility analysis (*n* = 1 study)

The cross-area comparability of QALYs is regarded by Bergmo^26^ as an advantage for cost-utility analyses (CUAs) over CEAs. Nevertheless, Bergmo also recognized that the typical estimation of QALYs using HRQoL utility values can be a limitation where nonhealth effects are relevant, as in DHIs (e.g., changes in access to services, time management, and health care provision).^26^

As a part of any of the above frameworks, the use of the net benefit regression framework (NBRF) can provide a platform to develop sensitivity analysis, as discussed by LeFevre et al.^35^ Within the NBRF, the sensitivity of the results can be tested against the maximum WTP amount for 1 additional QALY, obtaining a range of probabilities where a DHI may be more cost-effective than its alternatives. Investigating associations between subgroup differences (e.g., in gender, age, and ethnicity) and the net monetary benefit can reveal potential determinants of cost-effectiveness.^35^

##### Other frameworks (*n* = 4 studies)

As outlined by McNamee et al.,^22^ agent-based modeling can capture the complex (i.e., multifaceted behaviors are assumed by those delivering or receiving the intervention) and time-changing (e.g., individuals adapt and learn from previous experience) components of DHIs. In this framework, individuals follow nonlinear and adaptive behavior rules that reflect how decisions are taken autonomously and collectively in the context of DHIs.

McIntosh and Cairns^6^ discussed the use of conjoint analysis, where DHI users determine the relative importance of different levels of the features of the interventions through pairwise choices. These features relate not only to health outcomes but also to nonhealth and process outcomes,^6^ which can be central in digital health.

Kolasa and Kozinski^13^ delved into the use of multicriteria decision making, where the multifaceted features of digital health are explored, as weights are assigned to the (at times conflicting) preferences elicited from the different stakeholders.

Lastly, Le Goff-Pronost and Sicotte^27^ presented a 5-step framework for economic evaluations of DHIs, where 1) a traditional economic evaluation is integrated with longitudinal and stakeholder analyses, 2) a break-even point measures the volume of services needed to cover the fixed costs, 3) a net present value is calculated to discount future costs and consequences, 4) social benefits are estimated (e.g., network effects whereby the entry of new users increases the network’s overall value), and 5) sensitivity analyses test the impact of different factors on the results.

#### Instruments to collect health states data and techniques used to elicit utility values

While the use of the EuroQol Five Dimension (EQ-5D) descriptive system in economic evaluations of DHIs is recommended in methodological guidelines,^37^ generic HRQoL instruments may not be suitable to measure nonhealth effects of DHIs, as underlined by Mistry^7^ and Bongiovanni-Delarozière and Le Goff-Pronost.^34^ Moreover, Bergmo^25^ warned that, given the different eliciting techniques available, different utility values for similar health states may arise.^25^ For example, McIntosh and Cairns^6^ recommended the WTP method to elicit utility values, but Snoswell et al.^12^ recognized that different ways to ask the WTP from digital health users (e.g., multiple-choice or open-ended questions) may influence the responses and corresponding utility values. Overall, users need to see the full picture of what they are valuing,^12^ which includes the changing nature of DHIs and the range of services or effects produced (health and nonhealth outcomes).

Discrete choice experiments (DCEs) could reflect this dynamic nature. According to Snoswell et al.,^12^ the DCE tradeoff questions allow users to make choices around variations of DHIs, creating a preference-based ranking of the different aspects and characteristics (e.g., waiting time, clinical interaction, technological options) that form the overall WTP value.^12^

#### Generic outcome measures

Because DHIs can trigger indirect effects on health outcomes, as pointed out by McIntosh and Cairns,^6^ Ohinmaa et al.^23^ indicated that the use of QALYs and other generic outcome measures could miss shorter-term and process outcomes that are still important in digital health.

Bergmo^25^ explained how the estimation of QALYs through generic HRQoL instruments, like the EQ-5D, may miss disease-specific factors of relevance or underestimate the value of interventions for people whose improvements in health status or life expectancy will not be substantial. Bergmo^26^ also underlined that in digital health, other impacts could be relevant, for example, how digital health users feel secure and empowered.

Moreover, Kolasa and Kozinski^13^ argued that the typical HRQoL-based estimation of QALYs ignores the perspectives of digital health stakeholders beyond the patients (e.g., clinicians, health care managers, and funding bodies) and may fail to capture the full value of clinical and organizational effects.^13^

#### Disease-specific outcome measures

As disease-specific outcome measures may better capture the health-related effects triggered by DHIs on users, the common criticism over their lack of cross-area comparability is nevertheless echoed in the DHI field (see Bergmo^25^).

As with generic outcome measures, incorporating indirect effects of DHIs can be complex when using disease-specific outcome measures. For example, Davalos et al.^5^ explained how identifying and measuring the benefits of DHIs that indirectly help improve medication adherence is not straightforward, even if the subsequent effects on patients’ outcomes may seem apparent.

### Supplementary Narrative Synthesis on Costs and Nonhealth Outcomes

By presenting the above arguments, we focused on the traditional methodological areas that characterize the analytical frameworks and the measurement of outcomes in economic evaluations of any health care intervention. Nevertheless, we recognize that other methodological areas are important in economic evaluations in general, such as, how to measure and value costs, and in economic evaluations of DHIs in particular, how to measure and value nonhealth outcomes. As such, we also explored whether the included studies provided any arguments on how to measure and value costs and nonhealth outcomes in a supplementary narrative synthesis included in Appendix S1. Despite the assessment of costs being out of the scope of our systematic review, we believe that this supplementary narrative synthesis enriches our review by providing evidence on how to capture the wide range of costs and consequences triggered by DHIs.

## Discussion

### Place in the Literature

To our knowledge, this is the first systematic review to investigate how the effects of DHIs could be measured in economic evaluations. Reviews such as those by Rojas and Gagnon^38^ and Bergmo^28^ identified indicators for costs and effectiveness used to assess telemedicine interventions and commented on the lack of a common set of indicators that would facilitate cross-area comparability. However, unlike our review, those reviews did not evaluate the suitability of the analytical frameworks and outcome measures in economic evaluations of DHIs and therefore were not included in our final analysis. Another review by Bergmo,^25^ included in our review, explored the use of health state utilities to generate QALYs and transparency of methods in economic evaluations of DHIs. Jankovic et al.^39^ discussed the significance of the perspective for the identification of outcomes and the lack of clear tradeoffs between health gains and costs when disease-specific outcome measures are used. Kolasa and Kozinski,^13^ also included in our review, developed recommendations on how the value assessment of DHIs should be carried out, recognizing that QALYs may not be appropriate to capture the multidimensional character of DHIs. Lastly, an ongoing systematic review by Hariz et al.^40^ is set to identify the methodological choices made in economic evaluations of internet-based eHealth interventions (e.g., time horizon, perspective, choice of costs and outcomes) and to assess the impact of these choices on the results of economic evaluations.

Despite the useful findings of these systematic reviews, their inclusion criteria are limited to a few study designs, such as applied economic evaluations or guidelines. This narrow scope limits the number and range of findings obtained. Our review’s scope was more inclusive, as we also considered studies with a theoretical or methodological design. Our focus was not on identifying *which analytical frameworks and outcome measures were used* within the DHI economic evaluations but on identifying, assessing, and summarizing arguments on *how analytical frameworks and outcome measures could be used*, which gives our systematic review a more methodological basis. Compared with previous studies, we intended to provide a more in-depth discussion around the choices needed to measure the effects of DHIs. In this sense, we use our findings to formulate a set of recommendations that aims to help researchers in designing economic evaluations of DHIs. Similar tools exist in the literature, such as the flowchart proposed by LeFevre et al.^35^ for the economic evaluations of any health care interventions or the recommendations proposed by Kolasa and Kozinski^13^ for the economic evaluations of DHIs. Compared with LeFevre et al.,^35^ our recommendations focus specifically on economic evaluations of DHIs while, compared with Kolasa and Kozinski^13^ who systematically reviewed DHIs guidelines, we base our recommendations on a wider evidence base. Our recommendations intend to address researchers’ challenges in designing economic evaluations. However, they are not prescriptive nor represent a one-size-fits-all approach. On the contrary, they are an aiding tool in which the suggested analyses and tasks can be adapted to (or even excluded in consideration of) the individual DHI context (specific health area, setting, and type of decision maker), time frame for DHI implementation, and resources devoted to a specific economic evaluation.

### Set of Recommendations for Measuring Effects of DHIs in Economic Evaluations

**Figure 2:**
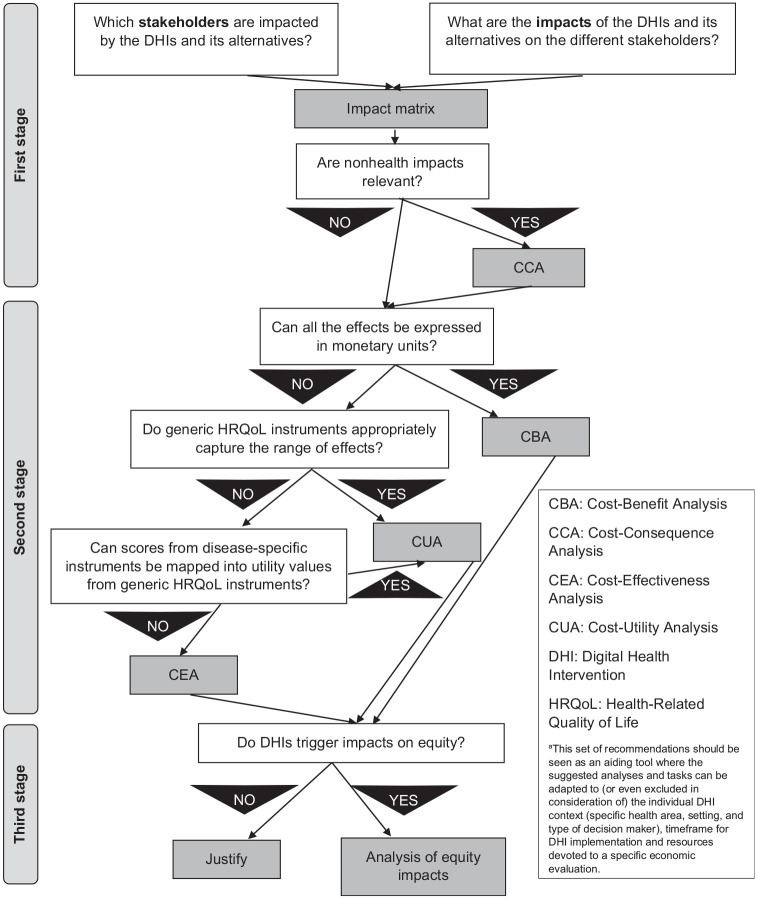
Flow chart summarizing the set of recommendations for measuring effects of digital health interventions (DHIs) in economic evaluations.^a^

Below we describe our 3-stage set of recommendations, which is illustrated in Figure 2.

#### Development of the impact matrix and CCA

Given the multidimensional effects of DHIs, we suggest the use of a matrix to list these potential effects. For example, in Le Goff-Pronost and Sicotte^27^ and Bongiovanni-Delarozière and Le Goff-Pronost,^34^ impact matrices reveal the expected effects of DHIs on different stakeholders (e.g., patients and caregivers, health care professionals and institutions, governments) in terms of accessibility, organization, quality and safety of care, and costs.^41^

This could be a preparatory activity that helps develop a CCA, the analysis suggested by NICE when DHIs affect nonhealth outcomes.^37^ The CCA would present the expected effects as listed in the impact matrix for the DHI and its competing alternatives, together with their measurement in natural or monetary units.^6^

#### Incorporation of outcome measures in economic evaluations

The CCA could then be used to prepare a more methodologically complex economic evaluation. However, a consensus seems lacking on which analytical framework would best suit an economic evaluation of DHIs. The issues around converting outcomes in monetary units in CBAs,^5,34^ the lack of generalizability of area-specific outcomes of interest in CEAs,^24^ and the limited ability of outcome measures estimating healthy years (typically QALYs) in capturing all relevant effects in CUAs are challenges that should be considered.^26^

Similarly, there does not seem to be a consensus on which outcome measures could be used. The use of QALYs in economic evaluations of health care interventions is backed by methodological guidelines,^42^ but their use has been debated in digital health.^13^ However, arguments favoring the use of alternative outcome measures are lacking in the digital health literature. The typical arguments against the use of QALYs seem to focus on the limited ability of HRQoL instruments, such as the EQ-5D, to capture a wider range of effects. Theoretically, the QALY construct ensures flexibility in terms of the dimensions that could be included in the underlying social welfare function, which may include nonhealth dimensions too, but this is somewhat unexplored in practice.^43^ The use of disease-specific outcome measures may help in capturing area-specific dimensions and effects that generic HRQoL instruments may miss. To increase the generalizability of the findings, mapping algorithms can be used to convert the scores obtained from disease-specific outcome measure into EQ-5D utility values.^44^ Direct methods to elicit utility values have also been discussed in the literature. For instance, DCEs could estimate the values attached to variations in the features of DHIs (e.g., different levels of access to health care services or health information received) to find the most valued combination by users.^12^

#### Assessment of impacts on equity

With their application to multiple health areas, DHIs naturally share equity-related concerns that are common in other health care interventions. However, some equity concerns can be considered specific to DHIs. For example, DHIs may facilitate access to health care services for people with existing limited access (e.g., those living in remote areas).^5^ At the same time, reaching familiarity with DHIs may not be straightforward for all users, and the lack of face-to-face interaction with health care professionals may depersonalize the provision of health care.^9^ Health care settings may differ on how receptive they are or how much they can invest in DHIs, which could limit a widespread geographical implementation. Consequently, existing health inequalities may potentially be widened by the introduction of DHIs.

Where possible, we encourage the use of extended CEAs to integrate traditional economic evaluations with an investigation of how equitable the distribution of the costs and effects of DHIs is.^35^ This can be carried out by formally analyzing the effects of DHIs on different subgroups through the NBRF, exploring the role played by socioeconomic, educational and clinical differences.^35^

Some recommendations (e.g., impact matrices and CCAs) are encouragingly shared by Gomes et al.^30^ Distinctively, our recommendations emerge from a systematic approach and cover more elements, such as utility values and equity impacts. To flesh out how to operationalize the recommendations, we built a case study presenting separate examples from studies that adopted approaches in line with the 3 stages above (Appendix S2).

### Strength and Limitations

The primary strength of our systematic review is the identification, assessment, and synthesis of arguments on how to measure the effects of DHIs in economic evaluations which, to our knowledge, represents a first attempt in the literature. Moreover, we used our findings to inform a 3-stage set of recommendations that can help practitioners in designing economic evaluations in this field.

One limitation lies in the underlying structural problem of systematic review processes, which are always prone to miss relevant studies. However, we believe that, by integrating our initial searches with backward and forward citation searching and gray literature searches, we are likely to have identified the relevant studies.

In this review, we focused on the analytical frameworks and outcome measures used in economic evaluations of DHIs, specifically looking at ways that have been used to try and overcome the limitations of using traditional approaches (e.g., HRQoL-informed QALYs). We recognize that other aspects of economic evaluations are potentially important and were not investigated here as out of our scope, such as the choice of the time horizon and modeling techniques. Similarly, our review was not specifically designed to search for studies including arguments on the identification and measurement of costs or on the choice of perspective (e.g., consideration of nonhealth outcomes). We did synthesize the arguments found from our included studies on costs and nonhealth outcomes in Appendix S1 to supplement our narrative synthesis.

Lastly, the generalizability of our proposed set of recommendations may be limited as DHIs tend to be applied to multiple health areas with diverse characteristics. However, we believe that our set of recommendations also addresses some of the issues inherent in DHIs, such as the multidimension of outcomes, which could be assessed using impact matrices and analyses of equity impacts, as suggested.

### Further Research

How to best measure outcomes in economic evaluations of DHIs is not straightforward, as specific features of digital health may make the application of traditional economic evaluation methods not suitable. Future research may focus on providing general guidance for DHI evaluations along the lines of our set of recommendations as well as specific guidance for health areas that are likely to trigger different effects (e.g., teleradiology v. telepsychiatry). Applying this guidance on ad hoc economic evaluations will prove useful too (as in Gomes et al.^30^).

Moreover, one of the key takeaways of our review is that no analytical framework nor outcome measure on their own may be able to fully capture the effects of DHIs. Future research may explore how a combination of different analytical approaches and outcome measures could be operationalized.

## Conclusions

The effects of DHIs can be varied and can go beyond the health outcomes of their users. In this systematic review, we searched for arguments on how these varied effects of DHIs could be measured in economic evaluations. The findings indicate that traditional frameworks (such as CBAs, CEAs or CUAs) and commonly used outcome measures (such as QALYs) may not appropriately determine the full value of DHIs.^13^

We used these findings to develop a 3-stage set of recommendations. Using impact matrices to list the multidimensional effects of DHIs on different stakeholders, and developing analyses to capture the equity impacts, can enrich traditional economic evaluations based on the estimation of cost-effectiveness. Despite the lack of generalizability that hinders economic evaluations in digital health,^5^ we believe that the recommendations could help the design of future economic evaluations in this field.

## Supplemental Material

sj-docx-1-mdm-10.1177_0272989X221132741 – Supplemental material for Analytical Frameworks and Outcome Measures in Economic Evaluations of Digital Health Interventions: A Methodological Systematic Review Click here for additional data file. Supplemental material, sj-docx-1-mdm-10.1177_0272989X221132741 for Analytical Frameworks and Outcome Measures in Economic Evaluations of Digital Health Interventions: A Methodological Systematic Review by Valerio Benedetto, Luís Filipe, Catherine Harris, Joseph Spencer, Carmel Hickson and Andrew Clegg in Medical Decision Making
