# Supplementary material for: Analytical Frameworks and Outcome Measures in Economic Evaluations of Digital Health Interventions: A Methodological Systematic Review
Source: Med Decis Making. 2022 Oct 19;43(1):125–38. doi: 10.1177/0272989X221132741 (PMC9742632; doi:10.1177/0272989X221132741)
Supplement: sj-docx-1-mdm-10.1177_0272989X221132741 – Supplemental material for Analytical Frameworks and Outcome Measures in Economic Evaluations of Digital Health Interventions: A Methodological Systematic Review [file sj-docx-1-mdm-10.1177_0272989X221132741.docx]

# Supplementary material

## Table S1. Preferred Reporting Items for Systematic Reviews and Meta-Analyses (PRISMA) 2020 Checklist^1^

| **Section and Topic** | **Item #** | **Checklist item** | **Location where item is reported** |
| --- | --- | --- | --- |
| **TITLE** | | |  |
| Title | 1 | Identify the report as a systematic review. | Title |
| **ABSTRACT** | | |  |
| Abstract | 2 | See the PRISMA 2020 for Abstracts checklist. | Abstract |
| **INTRODUCTION** | | |  |
| Rationale | 3 | Describe the rationale for the review in the context of existing knowledge. | Introduction |
| Objectives | 4 | Provide an explicit statement of the objective(s) or question(s) the review addresses. | Introduction |
| **METHODS** | | |  |
| Eligibility criteria | 5 | Specify the inclusion and exclusion criteria for the review and how studies were grouped for the syntheses. | Methods: Study selection  Table S8 |
| Information sources | 6 | Specify all databases, registers, websites, organisations, reference lists and other sources searched or consulted to identify studies. Specify the date when each source was last searched or consulted. | Methods: Search strategy |
| Search strategy | 7 | Present the full search strategies for all databases, registers and websites, including any filters and limits used. | Tables S2 to S7 |
| Selection process | 8 | Specify the methods used to decide whether a study met the inclusion criteria of the review, including how many reviewers screened each record and each report retrieved, whether they worked independently, and if applicable, details of automation tools used in the process. | Methods: Study selection |
| Data collection process | 9 | Specify the methods used to collect data from reports, including how many reviewers collected data from each report, whether they worked independently, any processes for obtaining or confirming data from study investigators, and if applicable, details of automation tools used in the process. | Methods: Data extraction |
| Data items | 10a | List and define all outcomes for which data were sought. Specify whether all results that were compatible with each outcome domain in each study were sought (e.g. for all measures, time points, analyses), and if not, the methods used to decide which results to collect. | Methods: Data extraction |
|  | 10b | List and define all other variables for which data were sought (e.g. participant and intervention characteristics, funding sources). Describe any assumptions made about any missing or unclear information. | Methods: Data extraction |
| Study risk of bias assessment | 11 | Specify the methods used to assess risk of bias in the included studies, including details of the tool(s) used, how many reviewers assessed each study and whether they worked independently, and if applicable, details of automation tools used in the process. | Methods: Quality assessment |
| Effect measures | 12 | Specify for each outcome the effect measure(s) (e.g. risk ratio, mean difference) used in the synthesis or presentation of results. | Methods: Data synthesis |
| Synthesis methods | 13a | Describe the processes used to decide which studies were eligible for each synthesis (e.g. tabulating the study intervention characteristics and comparing against the planned groups for each synthesis (item #5)). | Methods: Data synthesis |
|  | 13b | Describe any methods required to prepare the data for presentation or synthesis, such as handling of missing summary statistics, or data conversions. | Methods: Data synthesis |
|  | 13c | Describe any methods used to tabulate or visually display results of individual studies and syntheses. | Methods: Data synthesis |
|  | 13d | Describe any methods used to synthesize results and provide a rationale for the choice(s). If meta-analysis was performed, describe the model(s), method(s) to identify the presence and extent of statistical heterogeneity, and software package(s) used. | Methods: Data synthesis |
|  | 13e | Describe any methods used to explore possible causes of heterogeneity among study results (e.g. subgroup analysis, meta-regression). | Methods: Data synthesis |
|  | 13f | Describe any sensitivity analyses conducted to assess robustness of the synthesized results. | Methods: Data synthesis |
| Reporting bias assessment | 14 | Describe any methods used to assess risk of bias due to missing results in a synthesis (arising from reporting biases). | Methods: Data synthesis |
| Certainty assessment | 15 | Describe any methods used to assess certainty (or confidence) in the body of evidence for an outcome. | Methods: Data synthesis |
| **RESULTS** | | |  |
| Study selection | 16a | Describe the results of the search and selection process, from the number of records identified in the search to the number of studies included in the review, ideally using a flow diagram. | Results: Search results  Figure 1 |
|  | 16b | Cite studies that might appear to meet the inclusion criteria, but which were excluded, and explain why they were excluded. | Table S10 |
| Study characteristics | 17 | Cite each included study and present its characteristics. | Results: Overall summary of included studies  Table 1 |
| Risk of bias in studies | 18 | Present assessments of risk of bias for each included study. | Results: Synthesis of arguments |
| Results of individual studies | 19 | For all outcomes, present, for each study: (a) summary statistics for each group (where appropriate) and (b) an effect estimate and its precision (e.g. confidence/credible interval), ideally using structured tables or plots. | Results: Overall summary of included studies Table 1 |
| Results of syntheses | 20a | For each synthesis, briefly summarise the characteristics and risk of bias among contributing studies. | Results: Synthesis of arguments Appendices S1 and S2 |
|  | 20b | Present results of all statistical syntheses conducted. If meta-analysis was done, present for each the summary estimate and its precision (e.g. confidence/credible interval) and measures of statistical heterogeneity. If comparing groups, describe the direction of the effect. | Results: Synthesis of arguments |
|  | 20c | Present results of all investigations of possible causes of heterogeneity among study results. | Results: Synthesis of arguments |
|  | 20d | Present results of all sensitivity analyses conducted to assess the robustness of the synthesized results. | Results: Synthesis of arguments |
| Reporting biases | 21 | Present assessments of risk of bias due to missing results (arising from reporting biases) for each synthesis assessed. | Results: Synthesis of arguments |
| Certainty of evidence | 22 | Present assessments of certainty (or confidence) in the body of evidence for each outcome assessed. | Results: Synthesis of arguments |
| **DISCUSSION** | | |  |
| Discussion | 23a | Provide a general interpretation of the results in the context of other evidence. | Discussion: Place in the literature |
|  | 23b | Discuss any limitations of the evidence included in the review. | Discussion: Strength and limitations |
|  | 23c | Discuss any limitations of the review processes used. | Discussion: Strength and limitations |
|  | 23d | Discuss implications of the results for practice, policy, and future research. | Discussion: Set of recommendations for measuring effects of DHIs in economic evaluations  Discussion: Further research |
| **OTHER INFORMATION** | | |  |
| Registration and protocol | 24a | Provide registration information for the review, including register name and registration number, or state that the review was not registered. | Methods |
|  | 24b | Indicate where the review protocol can be accessed, or state that a protocol was not prepared. | Methods |
|  | 24c | Describe and explain any amendments to information provided at registration or in the protocol. | NA |
| Support | 25 | Describe sources of financial or non-financial support for the review, and the role of the funders or sponsors in the review. | Funding  Methods: Role of funding source |
| Competing interests | 26 | Declare any competing interests of review authors. | Conflict of Interests |
| Availability of data, code and other materials | 27 | Report which of the following are publicly available and where they can be found: template data collection forms; data extracted from included studies; data used for all analyses; analytic code; any other materials used in the review. | Acknowledgments |

## Table S2. Search strategy used in Medline (Ovid)

| **Search number** | **Query** |
| --- | --- |
| 1 | exp Telemedicine/ |
| 2 | Telenursing/ |
| 3 | Therapy, Computer-Assisted/ |
| 4 | Computer-Assisted Instruction/ |
| 5 | (mobile health* or m-health or mhealth or mobile care).ti,ab,kw,kf. |
| 6 | (Ehealth or e-health or electronic health* or electronic care or e-rehab* or erehab* or e-care or ecare or e-consult* or econsult* or e-diagnos* or ediagnos* or e-medicine or emedicine or e-nurs* or enurs* or e-psych* or epsych* or e-therap* or etherap*).ti,ab,kw,kf. |
| 7 | (telehealth* or tele-health* or telenurs* or tele-nurs* or telemedicine or tele-medicine or teleeducation or tele-education or telecare or tele-care or Telepsych* or tele-psych* or Telemonitor* or tele-monitor* or teleprevention or tele-prevention or teleadvice or Tele-advice or Teleassist* or tele-assist* or Telebased or tele-based or Teletherapy or tele-therapy or Teleconsult* or tele-consult* or Teletriag* or tele-triag* or telerehab* or tele-rehab* or telecoaching or tele-coaching or telemanagement or tele-management or telepharmacy or tele-pharmacy or telesupport or tele-support or teledermatolog* or tele-dermatolog* or telehome* or tele-home* or telescreen* or tele-screen* or telediagnos* or tele-diagnos* or telematic*).ti,ab,kf,kw. |
| 8 | ((digital or online or on-line or virtual or computer* or web based or web delivered or internet) adj4 (health* or care or medicine or intervention* or therap* or treatment* or educat* or training)).tw. |
| 9 | ((Remote or digital or video or virtual or wireless or phone* or telephone* or smartphone*) adj4 (consult* or appointment* or conferenc* or triag* or care or monitor* or check up$1 or checkup$1 or support)).tw. |
| 10 | (Videoconferenc* or video chat* or video call* or instant messag* or sms or short messaging service or texting or phone messag*).tw. |
| 11 | or/1-10 |
| 12 | (electronic health record* or electronic care record*).ti,ab,kf,kw. |
| 13 | 11 not 12 |
| 14 | Health Care Costs/ |
| 15 | Telemedicine/ec [Economics] |
| 16 | Quality-Adjusted Life Years/ |
| 17 | (economic* adj6 (aspect* or model* or framework* or frame work* or method* or quality or technique* or outcome* or tool* or concept* or assess*)).tw. |
| 18 | ((preference or cost effectiveness or cost benefit) adj3 measur*).tw. |
| 19 | ((quality adjusted life year* or qaly*) and (alternative* or method* or utilit* or critici* or limitation* or drawback* or draw back* or problem* or disadvantage* or flaw* or weak* or issue* or disbenefit* or pitfall* or appropriate* or inappropriate* or suitab* or unsuitab*)).tw. |
| 20 | (quality adjusted life year* or qaly*).ti. |
| 21 | (quality of life adj3 (measur* or evaluat* or assess*)).tw. |
| 22 | (value based health or (valu* adj2 health state*)).tw. |
| 23 | (valu* adj3 health adj3 (outcome* or measur*)).tw. |
| 24 | or/14-23 |
| 25 | 13 and 24 |
| 26 | limit 25 to english language |

## Table S3. Search strategy used in Embase (Ovid)

| **Search number** | **Query** |
| --- | --- |
| 1 | exp telemedicine/ |
| 2 | telenursing/ |
| 3 | computer assisted therapy/ |
| 4 | (mobile health* or m-health or mhealth or mobile care).ti,ab,kw. |
| 5 | (Ehealth or e-health or electronic health* or electronic care or e-rehab* or erehab* or e-care or ecare or e-consult* or econsult* or e-diagnos* or ediagnos* or e-medicine or emedicine or e-nurs* or enurs* or e-psych* or epsych* or e-therap* or etherap*).ti,ab,kw. |
| 6 | (telehealth* or tele-health* or telenurs* or tele-nurs* or telemedicine or tele-medicine or teleeducation or tele-education or telecare or tele-care or Telepsych* or tele-psych* or Telemonitor* or tele-monitor* or teleprevention or tele-prevention or teleadvice or Tele-advice or Teleassist* or tele-assist* or Telebased or tele-based or Teletherapy or tele-therapy or Teleconsult* or tele-consult* or Teletriag* or tele-triag* or telerehab* or tele-rehab* or telecoaching or tele-coaching or telemanagement or tele-management or telepharmacy or tele-pharmacy or telesupport or tele-support or teledermatolog* or tele-dermatolog* or telehome* or tele-home* or telescreen* or tele-screen* or telediagnos* or tele-diagnos* or telematic*).ti,ab,kw. |
| 7 | ((digital or online or on-line or virtual or computer* or web based or web delivered or internet) adj4 (health* or care or medicine or intervention* or therap* or treatment* or educat* or training)).tw. |
| 8 | ((Remote or digital or video or virtual or wireless or phone* or telephone* or smartphone*) adj4 (consult* or appointment* or conferenc* or triag* or care or monitor* or check up$1 or checkup$1 or support)).tw. |
| 9 | (Videoconferenc* or video chat* or video call* or instant messag* or sms or short messaging service or texting or phone messag*).tw. |
| 10 | or/1-9 |
| 11 | (electronic health record* or electronic care record*).ti,ab,kw. |
| 12 | 10 not 11 |
| 13 | "health care cost"/ |
| 14 | (exp telemedicine/ or telenursing/ or computer assisted therapy/) and ec.fs. |
| 15 | quality adjusted life year/ |
| 16 | (economic* adj6 (aspect* or model* or framework* or frame work* or method* or quality or technique* or outcome* or tool* or concept* or assess*)).tw. |
| 17 | ((preference or cost effectiveness or cost benefit) adj3 measur*).tw. |
| 18 | ((quality adjusted life year* or qaly*) and (alternative* or method* or utilit* or critici* or limitation* or drawback* or draw back* or problem* or disadvantage* or flaw* or weak* or issue* or disbenefit* or pitfall* or appropriate* or inappropriate* or suitab* or unsuitab*)).tw. |
| 19 | (quality adjusted life year* or qaly*).ti. |
| 20 | (quality of life adj3 (measur* or evaluat* or assess*)).tw. |
| 21 | (value based health or (valu* adj2 health state*)).tw. |
| 22 | (valu* adj3 health adj3 (outcome* or measur*)).tw. |
| 23 | or/13-22 |
| 24 | 12 and 23 |
| 25 | limit 24 to english language |

## Table S4. Search strategy used in the Cochrane Database of Systematic Reviews and Cochrane Central Register of Controlled Trials (Cochrane Library)

| **Search number** | **Query** |
| --- | --- |
| 1 | MeSH descriptor: [Telemedicine] explode all trees |
| 2 | MeSH descriptor: [Telenursing] explode all trees |
| 3 | MeSH descriptor: [Therapy, Computer-Assisted] explode all trees |
| 4 | MeSH descriptor: [Computer-Assisted Instruction] explode all trees |
| 5 | ((mobile NEXT health*) or "m-health" or mhealth or "mobile care"):ti,ab,kw |
| 6 | (Ehealth or "e-health" or (electronic NEXT health*) or "electronic care" or "e-rehab" or "e-rehabilitation" or erehab* or "e-care" or ecare or "e-consultations" or "e-consulting" or econsult* or "e-diagnosis" or ediagnos* or "e-medicine" or emedicine or "e-nursing" or enurs* or "e-psychiatry" or "e-psychology" or epsych* or "e-therapy" or "e-therapies" or etherap*):ti,ab,kw |
| 7 | (telehealth* or (tele NEXT health*) or telenurs* or "tele-nursing" or telemedicine or "tele-medicine" or teleeducation or "tele-education" or telecare or "tele-care" or Telepsych* or "tele-psychiatry" or "tele-psychology" or Telemonitor* or "tele-monitoring" or teleprevention or "tele-prevention" or teleadvice or "Tele-advice" or Teleassist* or "tele-assisting" or "tele assistance" or Telebased or "tele-based" or Teletherapy or "tele-therapy" or Teleconsult* or "tele-consultations" or "tele-consulting" or Teletriag* or "tele-triage" or telerehab* or "tele-rehabilitation" or "tele-rehab" or telecoaching or "tele-coaching" or telemanagement or "tele-management" or telepharmacy or "tele-pharmacy" or telesupport or "tele-support" or teledermatolog* or "tele-dermatology" or telehome* or (tele NEXT home*) or telescreen* or "tele-screening" or telediagnos* or "tele-diagnosis" or telematic*):ti,ab,kw |
| 8 | ((digital or online or "on-line" or virtual or computer* or "web based" or "web delivered" or internet) NEAR/4 (health* or care or medicine or intervention* or therap* or treatment* or educat* or training)):ti,ab,kw |
| 9 | ((Remote or digital or video or virtual or wireless or phone* or telephone* or smartphone*) NEAR/4 (consult* or appointment* or conferenc* or triag* or care or monitor* or "check up" or "check ups" or checkup* or support)):ti,ab,kw |
| 10 | (Videoconferenc* or (video NEXT chat*) or (video NEXT call*) or (instant NEXT messag*) or sms or "short messaging service" or texting or (phone NEXT messag*)):ti,ab,kw |
| 11 | {OR #1-#10} |
| 12 | ("electronic health record" or "electronic care record" or "electronic health records" or "electronic care records"):ti,ab,kw |
| 13 | #11 NOT #12 |
| 14 | MeSH descriptor: [Health Care Costs] explode all trees |
| 15 | MeSH descriptor: [Telemedicine] explode all trees and with qualifier(s): [economics - EC] |
| 16 | MeSH descriptor: [Quality-Adjusted Life Years] explode all trees |
| 17 | (economic* NEAR/6 (aspect* or model* or framework* or frame work* or method* or quality or technique* or outcome* or tool* or concept* or assess*)):ti,ab,kw |
| 18 | ((preference or "cost effectiveness" or "cost benefit") NEAR/3 measur*):ti,ab,kw |
| 19 | (("quality adjusted life year" or "quality adjusted life years" or qaly*) and (alternative* or method* or utilit* or critici* or limitation* or drawback* or "draw back" or "draw backs" or problem* or disadvantage* or flaw* or weak* or issue* or disbenefit* or pitfall* or appropriate* or inappropriate* or suitab* or unsuitab*)):ti,ab,kw |
| 20 | ("quality adjusted life year" or "quality adjusted life years" or qaly*):ti |
| 21 | ("quality of life" NEAR/3 (measur* or evaluat* or assess*)):ti,ab,kw |
| 22 | ("value based health"):ti,ab,kw |
| 23 | (valu* NEAR/2 ("health state" or "health states")):ti,ab,kw |
| 24 | (valu* NEAR/3 health NEAR/3 (outcome* or measur*)):ti,ab,kw |
| 25 | {OR #14–#24} |
| 26 | #13 AND #25 |

## Table S5. Search strategy used in the International Health Technology Assessment Database

| **Query** |
| --- |
| (("Telenursing"[mh]) OR ("Telemedicine"[mhe])) AND (((valu* AND health AND (outcome* or measur*))[abs]) OR ("value based health") OR ("quality of life" AND (measur* or evaluat* or assess*)) OR ("quality adjusted life year*" or qaly*) OR (((preference or "cost effectiveness" or "cost benefit") AND measur*)[abs]) OR (economic* AND (aspect* or model* or framework* or frame work* or method* or quality or technique* or outcome* or tool* or concept* or assess*)) OR ("Quality-Adjusted Life Years"[mh]) OR ("Health Care Costs"[mh])) |
| (("mobile care") OR (mhealth) OR ("mobile health")) AND (((valu* AND health AND (outcome* or measur*))[abs]) OR ("value based health") OR ("quality of life" AND (measur* or evaluat* or assess*)) OR ("quality adjusted life year*" or qaly*) OR (((preference or "cost effectiveness" or "cost benefit") AND measur*)[abs]) OR (economic* AND (aspect* or model* or framework* or frame work* or method* or quality or technique* or outcome* or tool* or concept* or assess*)) OR ("Quality-Adjusted Life Years"[mh]) OR ("Health Care Costs"[mh])) |
| (Ehealth or "electronic health*" or "electronic care" or erehab* or ecare or econsult* or ediagnos* or emedicine or enurs* or epsych* or etherap*) AND (((valu* AND health AND (outcome* or measur*))[abs]) OR ("value based health") OR ("quality of life" AND (measur* or evaluat* or assess*)) OR ("quality adjusted life year*" or qaly*) OR (((preference or "cost effectiveness" or "cost benefit") AND measur*)[abs]) OR (economic* AND (aspect* or model* or framework* or frame work* or method* or quality or technique* or outcome* or tool* or concept* or assess*)) OR ("Quality-Adjusted Life Years"[mh]) OR ("Health Care Costs"[mh])) |
| (telehealth* or "tele-health*" or telenurs* or "tele-nurs*" or telemedicine or "tele-medicine" or teleeducation or "tele-education" or telecare or "tele-care" or Telepsych* or "tele-psych*" or Telemonitor* or "tele-monitor*" or teleprevention or "tele-prevention" or teleadvice or "Tele-advice" or Teleassist* or "tele-assist*" or Telebased or "tele-based" or Teletherapy or "tele-therapy" or Teleconsult* or "tele-consult*" or Teletriag* or "tele-triag*" or telerehab* or "tele-rehab*" or telecoaching or "tele-coaching" or telemanagement or "tele-management" or telepharmacy or "tele-pharmacy" or telesupport or "tele-support" or teledermatolog* or "tele-dermatolog*" or telehome* or "tele-home*" or telescreen* or "tele-screen*" or telediagnos* or "tele-diagnos*" or telematic*) AND (((valu* AND health AND (outcome* or measur*))[abs]) OR ("value based health") OR ("quality of life" AND (measur* or evaluat* or assess*)) OR ("quality adjusted life year*" or qaly*) OR (((preference or "cost effectiveness" or "cost benefit") AND measur*)[abs]) OR (economic* AND (aspect* or model* or framework* or frame work* or method* or quality or technique* or outcome* or tool* or concept* or assess*)) OR ("Quality-Adjusted Life Years"[mh]) OR ("Health Care Costs"[mh])) |
| (((digital or online or virtual or computer* or "web based" or "web delivered" or internet) AND (health* or care or medicine or intervention* or therap* or treatment* or educat* or training))[abs]) AND (((valu* AND health AND (outcome* or measur*))[abs]) OR ("value based health") OR ("quality of life" AND (measur* or evaluat* or assess*)) OR ("quality adjusted life year*" or qaly*) OR (((preference or "cost effectiveness" or "cost benefit") AND measur*)[abs]) OR (economic* AND (aspect* or model* or framework* or frame work* or method* or quality or technique* or outcome* or tool* or concept* or assess*)) OR ("Quality-Adjusted Life Years"[mh]) OR ("Health Care Costs"[mh])) |
| (((Remote or digital or video or virtual or wireless or phone* or telephone* or smartphone*) AND (consult* or appointment* or conferenc* or triag* or care or monitor* or "check up*" or checkup* or support))[abs]) AND (((valu* AND health AND (outcome* or measur*))[abs]) OR ("value based health") OR ("quality of life" AND (measur* or evaluat* or assess*)) OR ("quality adjusted life year*" or qaly*) OR (((preference or "cost effectiveness" or "cost benefit") AND measur*)[abs]) OR (economic* AND (aspect* or model* or framework* or frame work* or method* or quality or technique* or outcome* or tool* or concept* or assess*)) OR ("Quality-Adjusted Life Years"[mh]) OR ("Health Care Costs"[mh])) |
| ((Videoconferenc* or "video chat*" or "video call*" or "instant messag*" or sms or "short messaging service" or texting or "phone messag*")[abs]) AND (((valu* AND health AND (outcome* or measur*))[abs]) OR ("value based health") OR ("quality of life" AND (measur* or evaluat* or assess*)) OR ("quality adjusted life year*" or qaly*) OR (((preference or "cost effectiveness" or "cost benefit") AND measur*)[abs]) OR (economic* AND (aspect* or model* or framework* or frame work* or method* or quality or technique* or outcome* or tool* or concept* or assess*)) OR ("Quality-Adjusted Life Years"[mh]) OR ("Health Care Costs"[mh])) |

## Table S6. Search strategy used in the NHS Economic Evaluation Database

| **Search number** | **Query** |
| --- | --- |
| 1 | MeSH DESCRIPTOR Health Care Costs EXPLODE ALL TREES |
| 2 | MeSH DESCRIPTOR Quality-Adjusted Life Years EXPLODE ALL TREES |
| 3 | (economic* NEAR6 (aspect* or model* or framework* or frame work* or method* or quality or technique* or outcome* or tool* or concept* or assess*)) IN NHSEED |
| 4 | ((preference or cost effectiveness or cost benefit) NEAR3 measur*) IN NHSEED |
| 5 | (("quality adjusted life year*" or qaly*) AND (alternative* or method* or utilit* or critici* or limitation* or drawback* or "draw back*" or problem* or disadvantage* or flaw* or weak* or issue* or disbenefit* or pitfall* or appropriate* or inappropriate* or suitab* or unsuitab*)) IN NHSEED |
| 6 | ("quality of life" NEAR3 (measur* or evaluat* or assess*)) IN NHSEED |
| 7 | ("value based health") IN NHSEED |
| 8 | (valu* NEAR2 "health state*") IN NHSEED |
| 9 | (valu* NEAR3 health NEAR3 (outcome* or measur*)) IN NHSEED |
| 10 | #1 OR #2 OR #3 OR #4 OR #5 OR #6 OR #7 OR #8 OR #9 |
| 11 | MeSH DESCRIPTOR Telemedicine EXPLODE ALL TREES |
| 12 | MeSH DESCRIPTOR Telenursing EXPLODE ALL TREES |
| 13 | ("mobile health*" or "m-health" or mhealth or "mobile care") IN NHSEED |
| 14 | (Ehealth or "e-health" or "electronic health*" or "electronic care" or "e-rehab*" or erehab* or "e-care" or ecare or "e-consult*" or econsult* or "e-diagnos*" or ediagnos* or "e-medicine" or emedicine or "e-nurs*" or enurs* or "e-psych*" or epsych* or "e-therap*" or etherap*) IN NHSEED |
| 15 | (telehealth* or "tele-health*" or telenurs* or "tele-nurs*" or telemedicine or "tele-medicine" or teleeducation or "tele-education" or telecare or "tele-care" or Telepsych* or "tele-psych*" or Telemonitor* or "tele-monitor*" or teleprevention or "tele-prevention" or teleadvice or "Tele-advice" or Teleassist* or "tele-assist*" or Telebased or "tele-based" or Teletherapy or "tele-therapy" or Teleconsult* or "tele-consult*" or Teletriag* or "tele-triag*" or telerehab* or "tele-rehab*" or telecoaching or "tele-coaching" or telemanagement or "tele-management" or telepharmacy or "tele-pharmacy" or telesupport or "tele-support" or teledermatolog* or "tele-dermatolog*" or telehome* or "tele-home*" or telescreen* or "tele-screen*" or telediagnos* or "tele-diagnos*" or telematic*) IN NHSEED |
| 16 | ((digital or online or "on-line" or virtual or computer* or "web based" or "web delivered" or internet) NEAR4 (health* or care or medicine or intervention* or therap* or treatment* or educat* or training)) IN NHSEED |
| 17 | ((Remote or digital or video or virtual or wireless or phone* or telephone* or smartphone*) NEAR4 (consult* or appointment* or conferenc* or triag* or care or monitor* or "check up*" or checkup* or support)) IN NHSEED |
| 18 | (Videoconferenc* or "video chat*" or "video call*" or "instant messag*" or sms or "short messaging service" or texting or "phone messag*") IN NHSEED |
| 19 | #11 OR #12 OR #13 OR #14 OR #15 OR #16 OR #17 OR #18 |
| 20 | #10 AND #19 |

## Table S7. Websites and search string used in the grey literature searches

| **Websites** | **Search string** |
| --- | --- |
| ispor.org | ("mobile health*" OR mhealth OR "mobile care" OR Ehealth OR erehabilitation OR erehab OR telehealth OR telehealthcare OR telemedicine OR telecare OR telenursing) |
| healtheconomics.org |  |
| ohe.org |  |

## Table S8. Screening tool

| **Analytical frameworks and outcome measures in economic evaluations of digital health interventions: a methodological systematic review** | |
| --- | --- |
| **Research aim:**  To systematically identify and assess studies which discuss the challenges, advantages and disadvantages associated to the use of outcome measures such as quality-adjusted life-year (QALY) and its alternatives in economic evaluations of digital health interventions (DHIs) | |
| **Key elements** | |
| **Population** | **Included:**   - General population (i.e. patient or user) and healthcare professionals using a digital health (or similar) intervention. |
|  | **Excluded:**   - General population (i.e. patient or user) and healthcare professionals not using a digital health (or similar) intervention. |
| **Interventions** | **Included**:   - Any DHI, i.e. delivered using technologies facilitating a remote access and use, and adopted for the purpose of health promotion or improvement, and disease prevention and/or treatment. |
|  | **Excluded**:   - Any non-DHI. |
| **Settings** | **Any** |
| **Outcomes** | **Included:**   - Any study including a discussion of the theoretical and empirical challenges, advantages and disadvantages associated with the measurement, valuation and use of outcome measures (e.g. QALY and its alternatives), including the choice of analytical frameworks, for economic evaluations of DHIs. |
|  | **Excluded:**   - Any study that does not include a discussion of the methodological challenges attached to the use of analytical frameworks and outcome measure for economic evaluations of DHIs (e.g. studies which only list or summarise the outcome measures used in economic evaluations). |
| **Comparison** | **Any** (e.g. face-to-face or other DHIs) |
| **Study design** | **Included:**   - Empirical studies: - systematic reviews (and meta-analyses) of economic evaluations; - scoping reviews of economic evaluations; - qualitative reviews of economic evaluations; - narrative reviews of economic evaluations; - full applied economic evaluations (i.e. which consider both the costs and the consequences of DHIs and their comparators); - observational studies (e.g. cohort and case-control studies); - interventional studies (e.g. pre-post study, non-randomised and randomised controlled trials); - Non-empirical studies: - theoretical or conceptual papers; - economic guidelines and checklists; - position papers; - editorials; - commentaries - letters. |
|  | **Excluded**:   - Abstracts. |
| **Limits** | English language studies only. |

## Table S9. Guidance for Reporting Involvement of Patients and Public (GRIPP2) table^3^

| **Section and topic** | **Item** |
| --- | --- |
| 1: Aim of the study | To identify, assess and synthesise the arguments in the literature on how the effects of digital health interventions (DHIs) could be measured in economic evaluations. |
| 2: PPI methods | One public adviser (CHi) was recruited following the circulation of an expression of interest call within the Public Advisers’ Forum of the National Institute for Health and Care Research Applied Research Collaboration North West Coast (NIHR ARC NWC).  In the call we specified the aim, outline and timescales of the planned systematic review, together with the expected input from the public adviser. In particular, we sought input in terms of reading, reviewing and commenting on the draft protocol and report from the systematic review to inform the presentation, interpretation and application of the results.  The call also detailed the type of payment for the public adviser’s input. |
| 3: PPI results | Following the recruitment, an initial meeting between one of the co-authors (VB) and CHi was held, where the systematic review was discussed, and reciprocal expectations set out in more detail. The tasks and related time commitment for CHi, as well as the support available from the other co-authors, were outlined and agreed.  After the initial meeting, CHi provided feedback to the draft protocol of the systematic review. Valuable comments were provided with focus on the assessment of health inequalities which may arise from the implementation of DHIs (e.g. lack of confidence in using a new technology). This feedback was incorporated in the protocol which was then registered on PROSPERO, with CHi included as one of the co-authors.  During the systematic review process, one of the co-authors (VB) sent regular updates to CHi to provide information about the progress of the work and any adjustments in the expected timescale.  Once the draft of the manuscript was reviewed by the other co-authors, it was then sent to CHi for feedback. CHi found the descriptions of the systematic review process to be very thorough and clear in terms of the search strategy and literature review. On the other hand, the discussion of analytical frameworks was deemed quite complex due to the use of technical economics language and acronyms. CHi appreciated the inclusion of a specific section dedicated to equity impacts, given her concerns that the increase in digital health provision could exclude disadvantaged and elderly patients, thus potentially contributing to the widening of health inequalities. Lastly, CHi found the draft clear in outlining the potential for further research in light of the increasing use of digital health initiatives and possibility of using a combination of analytical frameworks to measure their outcomes.  CHi also reviewed this table, agreeing with the content describing her involvement and the PPI process. |
| 4: Discussion and conclusions | The PPI activities contributed to enhance the systematic review process mainly in two ways. First, CHi provided useful feedback on areas where the synthesis of the findings should focus on, in particular on where differences across digital health users may emerge (e.g. geographical and prior knowledge variation). These differences are likely to trigger health inequalities which may not be necessarily captured by traditional economic evaluations, which focus on an assessment of cost-effectiveness but should incorporate equity assessments too, as we recommend in our proposed set of recommendations on how to measure outcomes of DHIs in economic evaluations.  Second, the collaboration with CHi helped us focus on the clarity of the description of the systematic review process in the protocol and manuscript. We strived to minimise the use of technical terms or, where not possible, briefly explain them. However, this is an area where further work is needed, as CHi rightly pointed out in the review of the manuscript. While to some extent the use of technical terms and acronyms is almost inevitable in academic papers, we will certainly keep these concerns in mind when we will prepare presentations and lay summaries of our findings for audiences which are wider than the academic one. |
| 5: Reflections/critical perspective | With the systematic review being methodological in nature, it was our intention to facilitate the understanding of the process and findings of our review to both specialist and non-specialist audiences. Thanks to the input of CHi, we understood the strength and limitations of the manuscript draft. While the clear description of the systematic review process and the inclusion of a section specifically dedicated to equity impacts were well welcomed by CHi, her concerns over the use of acronyms and economic language deserve attention. We will certainly take these concerns on board when preparing presentations and lay summaries from our work. This is a lesson that we would recommend to any researcher involved in writing up and disseminating methodological findings.  In relation to the actual PPI process, we thought that clear and regular communication with the public adviser was going to be key throughout the process. As such, we set out our reciprocal expectations clearly from the outset and sent regular updates on our work to keep CHi abreast of our progress. We think it is important to maintain regular contact with the public adviser during the process especially when, as is the case for systematic reviews, the work can be particularly lengthy and subject to change compared with the initially planned timescales. More importantly, our aim was to generate frank discussion with the public adviser about the quality of the work. This can be fostered by finding ways to rise the engagement of the public adviser: regular contacts and creating an environment where everyone’s view is valued are some of the methods that we would recommend to other researchers developing PPI activities. |

PPI: Patient and Public Involvement.

## Table S10. Records excluded following full-text screening (n=43)

| **First author (year)** | **Reason for exclusion** | | | |
| --- | --- | --- | --- | --- |
|  | **No detail on measurement, valuation, choice and use of outcome measures for economic evaluations** | **Little or marginal detail on measurement, valuation, choice and use of outcome measures for economic evaluations** | **Full text not available in English** | **Full text not available** |
| Abimbola (2019)^4^ |  | 🗸 |  |  |
| Bashshur (1995)^5^ |  | 🗸 |  |  |
| Beecham (2019)^6^ |  | 🗸 |  |  |
| Berki (1975)^7^ |  |  |  | 🗸 |
| Bertoncello (2018)^8^ | 🗸 |  |  |  |
| Charrier (2016)^9^ | 🗸 |  |  |  |
| Crowe (1992)^10^ |  | 🗸 |  |  |
| Doze (1999)^11^ |  | 🗸 |  |  |
| Enam (2018)^12^ | 🗸 |  |  |  |
| Goldstein (2001)^13^ | 🗸 |  |  |  |
| Griscenko (2012)^14^ | 🗸 |  |  |  |
| Hailey (1999)^15^ |  | 🗸 |  |  |
| Hailey (2000)^16^ |  | 🗸 |  |  |
| Hailey (2003)^17^ |  | 🗸 |  |  |
| Hailey (2004)^18^ |  | 🗸 |  |  |
| Hakansson (2000)^19^ |  | 🗸 |  |  |
| Hughes (2002)^20^ | 🗸 |  |  |  |
| Iribarren (2017)^21^ | 🗸 |  |  |  |
| Jankovic (2021)^22^ |  | 🗸 |  |  |
| Jean (2016)^23^ |  |  | 🗸 |  |
| Jurkeviciute (2020)^24^ |  | 🗸 |  |  |
| Kadu (2019)^25^ |  | 🗸 |  |  |
| Kennedy (2005)^26^ |  | 🗸 |  |  |
| Kidholm (2010)^27^ |  | 🗸 |  |  |
| Kidholm (2012)^28^ |  | 🗸 |  |  |
| Kidholm (2017)^29^ | 🗸 |  |  |  |
| Kristiansen (2003)^30^ | 🗸 |  |  |  |
| Lobley (1997)^31^ |  | 🗸 |  |  |
| Luxton (2013)^32^ |  | 🗸 |  |  |
| Luzi (2016)^33^ |  | 🗸 |  |  |
| Mair (2000)^34^ |  | 🗸 |  |  |
| Ohinmaa (2002)^35^ |  | 🗸 |  |  |
| Phillips (2017)^36^ |  | 🗸 |  |  |
| Powell (2020)^37^ |  | 🗸 |  |  |
| Rojas (2008)^38^ |  | 🗸 |  |  |
| Ruckdäschel (2006)^39^ |  | 🗸 |  |  |
| Rudolph (2011)^40^ | 🗸 |  |  |  |
| Scott (2007)^41^ |  | 🗸 |  |  |
| Sisk (1998)^42^ |  | 🗸 |  |  |
| Suzuki (2019)^43^ | 🗸 |  |  |  |
| Vis (2020)^44^ | 🗸 |  |  |  |
| Wang (2014)^45^ |  | 🗸 |  |  |
| Zanaboni (2011)^46^ | 🗸 |  |  |  |
| **n(%)** | 13(30) | 28(65) | 1(2) | 1(2) |

## Appendix S1. Supplement to narrative synthesis: arguments from the included studies on the measurement of digital health interventions’ costs and non-health outcomes

In this appendix we summarise arguments, identified in the included studies, on how to measure costs and non-health outcomes. Therefore, this appendix focuses on those impacts which are not typically measured by generic and disease-specific outcome measures in economic evaluations, and complements the narrative synthesis presented in our main article.

It is important to note that we focus here on *how to measure costs and non-health outcomes* triggered by digital health interventions (DHIs)*,* rather than on listing *which costs and non-health outcomes* are triggered by DHIs. Such lists abound in the included studies and we refer the reader to the related articles (see for example the lists provided by Angjellari-Dajci et al., 2013^47^, Davalos et al., 2009^48^, and Le-Goff Pronost and Sicotte, 2010^49^).

It should also be noted that the cost side was not the focus of our systematic review, since our search strategy was specified to capture arguments on how to measure outcomes from DHIs. Consequently, the list of arguments on how to measure costs presented here is only illustrative and should not be seen as exhaustive.

Costs

The study by Bergmo (2015)^50^ delineated the standard method to measure and value healthcare costs in economic evaluations of DHIs. This method involves three steps where the different cost categories (such as labour, capital and overheads) are first identified, then measured in physical units (resource use), and finally valued using tariffs (unit costs).

Once the cost categories are identified, resource use data are usually sourced from medical records and case report forms. However, for their actual measurement two approaches can be adopted (in conjunction or separately). With micro-costing, costs are separated into individual components (for example, resource use for investigations, tests or medicines and so on). With gross costing instead, bundles of service use are considered (for example, by measuring bed days or hospital stays)^50^.

Then tariffs are applied to each resource use to obtain the total healthcare costs, as recommended by economic guidelines (for example, the guideline by the UK National Institute for Health and Care Excellence on DHIs (2018)^51^). Tariffs can be site-specific or system-specific, with the latter derived from national databases listing unit costs for labour, specific investigations, tests or medicines, diagnosis-related groups and health-resource groups^50^. Equipment costs should be also considered, and their costs annuitized over the lifetime of the equipment to account for depreciation and the opportunity cost of the capital invested^50^.

Among the indirect costs, the time taken off work due to illness or to seek care (as a patient), or to care for someone (as a caregiver), is also considered in the study by Bergmo (2015)^50^. Time off work is usually measured by calculating productivity losses. These are valued by referring to gross wages^48, 50^ or by adopting the friction cost method, which is based on the time needed to reinstate production at the level it would have been had the patient or caregiver carried on working^50^.

Non-healthcare outcomes

As indicated by Bergmo (2015), the measurement of non-healthcare outcomes, such as access to care, sense of empowerment and knowledge transfer may be challenging^50^. Among the included studies, perhaps the most significant source of information on how to measure non-health outcomes is the study by Davalos et al. (2009)^48^, which includes insights on monetising outcomes in the context of cost-benefit analyses (CBAs). Here we focus on some key non-health outcomes, but for more details on other outcomes the reader can refer directly to the article by Davalos et al. (2009)^48^.

For some of the non-health outcomes, the methods available to monetise them are rather straightforward. For example, for the reduced travel triggered by a DHI (for patients or users and healthcare professionals alike), the monetary value can be obtained by multiplying the distance to the healthcare site by the mileage allowance rate, or simply by referring to the specific fares of the mode of transport used^48^.

For other non-health outcomes, the associated methods of monetisation are more complex. For example, for knowledge transfer among healthcare professionals, Davalos et al. (2009)^48^ suggested the estimation of avoided referrals or, alternatively, the training time required to obtain the same knowledge together with the time taken off work to attend training. These would be then monetised by considering the healthcare professionals’ specific hourly wage (for the time not spent on the referral or, alternatively, for the time not spent on training and the consequent work time saved) and the patients’ avoided costs (for the avoided referrals). Where market values are not available, then a willingness-to-pay analysis may be useful to understand the monetary value placed by individuals on a specific DHI. However, separating out the individual values of specific outcomes (e.g. patient’s satisfaction or acquired health knowledge) remains difficult^48^.

## Appendix S2. Case study on set of recommendations on measuring effects of digital health interventions in economic evaluations: examples from published studies

In this case study, we present examples of published studies which show how our evidence-based recommendations on the measurement of outcomes in economic evaluations of digital health interventions can be operationalised.

### Development of impact matrix and Cost-Consequence Analysis (CCA)

#### Impact matrix

A fitting example of a comprehensive impact matrix based on multiple stakeholders and dimensions comes from the study by Le Goff-Pronost and Sicotte (2010)^49^. The authors designed a matrix which intended to describe the potential impacts of telemedicine networks for patients, physicians, hospitals and governments in terms of five attributes, namely: 1) accessibility; 2) acceptability; 3) quality; 4) organisation; and 5) costs and benefits.

Another similar example can be found in Bongiovanni-Delarozière and Le Goff-Pronost (2017)^52^. These authors followed the French National Authority for Health methodological framework in order to create an impact matrix based on a systematic review of economic evaluations of telemedicine interventions. This matrix is set to describe changes in: 1) accessibility; 2) professional practice/care organisation; 3) care quality/safety; and 4) costs. Again, the four attributes can be evaluated by taking into account the separate impacts on patients and family caregivers, physicians/allied health professionals, healthcare institutions, government, health insurance companies and local authorities.

#### CCA

The study by Snoswell et al. (2019)^53^ represents an example of how relevant non-health impacts could be captured and emphasised using CCAs. In this study telehealth was compared with other two modes of delivery to provide specialist clinics to remote Indigenous people with diabetes in Australia. Given the unique and diverse consequences triggered by each mode of delivery, the authors adopted a CCA in order to present the consequences separately, rather than using a composite outcome measure. Among the consequences analysed, non-health consequences for the patients were related to time (such as the time taken away from other activities and the waiting time for consultation), capacity (such as the number of patients that could access each clinic at one time) and place (such as the location of the appointments).

Another example where time-related consequences (on work and travel) were considered is in the study by Noble et al. (2005)^54^, whose CCA compared telemedicine and routine GPs appointments for the treatment of minor injuries in the UK.

### Incorporation of outcome measures in economic evaluations

#### Cost-Benefit Analysis (CBA)

Langabeer et al. (2017)^55^ performed a CBA of a telehealth-based consultation between patients and physicians working in Emergency Medical Services, where the need for patient transportation to a hospital emergency department was evaluated. Averted transportation costs for patients and averted emergency consultations costs were counted as benefits, and then compared against the costs triggered by the intervention. Since the intervention was examined only in terms of efficiency implications, implementing a CBA was a reasonable choice in this case.

However, if the focus also embraces the patients’ utility from the intervention, then a willingness-to-pay (WTP) analysis is an option that may need to be explored as part of a CBA. This was the case of the study by Stahl and Dixon (2010)^56^, where videoconferencing was compared with face-to-face office visits in primary care. After being examined in both settings, patients were asked about their WTP for videoconferencing, which can be computed based on open-ended questions, binary choices like standard gamble elicitation, or bidding.

Another more sophisticated option to estimate WTP is to use discrete choice experiments (DCEs). This method is more robust to typical stated preference problems, such as strategic answering or hypothetical bias. As an example, Chang et al. (2017)^57^ chose to use a DCE to evaluate the benefits obtained by households using online health services. In this DCE, a hypothetical internet network service was evaluated by incorporating both price and online health service elements as attributes, around which the users’ preferences were elicited.

#### Cost-Utility Analysis (CUA)

According to our evidence-based set of recommendations, CUAs can be adopted when generic health-related quality of life (HRQoL) instruments like the EuroQol Five Dimension descriptive system (EQ-5D) are able to capture the relevant range of effects triggered by the interventions under examination. This is the case of the CUA developed by Noben et al. (2017)^58^ who compared a web-based employability intervention against usual care (i.e. regular trade union support) for people with work-related disabilities in the Netherlands. Since the focus was on a range of disabilities rather than specific ones, using a generic and non-specific HRQoL instrument like the EQ-5D to then estimate quality-adjusted life years (QALYs) seems sensible.

The approach chosen in the study by Tan et al. (2021)^59^ on telestroke in China may be considered as an appropriate example for the use of CUA too. Importantly, the utility scores in this CUA, estimated using the EQ-5D, were assigned differently to patients categorised according to the severity of the consequences of their stroke, as measured by the modified Rankin Scale. The resulting QALYs were reflective of the different degrees of disease-specific disability or dependence of the stroke survivors.

#### Mapping algorithms

An example of the use of mapping algorithms in economic evaluations of digital health interventions is provided in the study by Naveršnik and Mrhar (2014)^60^ who evaluated a web-based depression intervention. The effectiveness of the intervention was measured using the Beck Depression Inventory scale, and the relative scores were then converted into QALY weights. As recognised by the authors, when interventions for people with mental disorders are assessed, generic HRQoL instruments like the EQ-5D may not be sensitive enough to capture the health and quality of life impacts of the patients. In this and other disease areas, existing^61^ or *de novo* mapping algorithms should be considered to generate disease-sensitive QALYs.

#### Cost-Effectiveness Analysis (CEA)

The previously mentioned study by Noben et al. (2017)^58^ represents an example of when a CUA and a CEA can co-exist. As the web-based intervention under examination aimed to improved employability of the users, their work ability was assessed in a CEA (in terms of cost per gain in work ability outcome), together with the QALYs analysed in the CUA.

In another CEA, Franzini et al. (2011)^62^ evaluated the effect of tele-intensive care unit programmes on patient mortality. As this outcome of interest could not be suitably measured by HRQoL instruments, a CEA based on the effects of the intervention on mortality was deemed adequate.

### Assessment of impacts on equity

As mentioned in LeFevre et al. (2017)^63^, the use of extended cost-effectiveness analyses to assess equity impacts is limited in economic evaluations. However, we have identified other ways to investigate equity impacts from digital health interventions in the literature. A fitting example comes from a trial which looked at investigating the cost-effectiveness of mHealth and community mobilisation interventions in preventing and controlling Type 2 diabetes mellitus and risk factors for non-communicable diseases in rural Bangladesh^64^. In this trial, the equity analysis was first set out in the protocol of the economic evaluation^65^ and then developed^66^. Sub-group analyses were intended to capture how the exposure to and the effects of the interventions differed across participants with different socio-economic characteristics, such as age, gender and wealth^66^.

Witt Udsen et al. (2017)^67^ provided another example of the use of sub-group analysis. In this study, the incremental QALYs resulting from a tele-healthcare trial for people with chronic obstructive pulmonary disease in Denmark were further investigated by stratifying the population according to their comorbidities, age, gender, prior social care resource use and delivery sites. In particular, geographical stratification is likely to play a key role in the delivery of digital health interventions, which may alter the access to healthcare services for underserved segments of the population (e.g. those living in rural areas). As such, techniques like geospatial assessment, as explored in a study on a virtual urgent care programme in the United States by Khairat et al. (2019)^68^, are worth considering as part of a wider analysis on equity impacts.

# References

1. Page MJ, McKenzie JE, Bossuyt PM, et al. The PRISMA 2020 statement: an updated guideline for reporting systematic reviews. *Bmj* 2021; 372: n71. 2021/03/31. DOI: 10.1136/bmj.n71.

2. Nagamitsu S, Horikoshi M, Sakashita K, et al. 1.7 Effectiveness of health promotion interventions for adolescents using health care visits and a smartphone CBT application: a randomized controlled trial. *Journal of the American Academy of Child and Adolescent Psychiatry* 2020; 59: S141‐. DOI: 10.1016/j.jaac.2020.08.033.

3. Staniszewska S, Brett J, Simera I, et al. GRIPP2 reporting checklists: tools to improve reporting of patient and public involvement in research. *Res Involv Engagem* 2017; 3: 13. 2017/10/25. DOI: 10.1186/s40900-017-0062-2.

4. Abimbola S, Keelan S, Everett M, et al. The medium, the message and the measure: A theory-driven review on the value of telehealth as a patient-facing digital health innovation. *Health Economics Review* 2019; 9. Review. DOI: 10.1186/s13561-019-0239-5.

5. Bashshur RL. Telemedicine effects: cost, quality, and access. *Journal of Medical Systems* 1995; 19: 81-91.

6. Beecham J, Bonin EM, Gorlich D, et al. Assessing the costs and cost-effectiveness of ICare internet-based interventions (protocol). *Internet Interventions* 2019; 16: 12-19. DOI: <https://dx.doi.org/10.1016/j.invent.2018.02.009>.

7. Berki SE. Telemedicine: some economic implications. *Telemedicine, Explorations in the use of telecommunications in health care Springfield, IL: Charles Thomas* 1975: 175-191.

8. Bertoncello C, Colucci M, Baldovin T, et al. How does it work? Factors involved in telemedicine home-interventions effectiveness: A review of reviews. *PLoS ONE* 2018; 13: e0207332. DOI: <https://dx.doi.org/10.1371/journal.pone.0207332>.

9. Charrier N, Zarca K, Durand-Zaleski I, et al. Efficacy and cost effectiveness of telemedicine for improving access to care in the Paris region: study protocols for eight trials. *BMC Health Services Research* 2016; 16: 45. DOI: <https://dx.doi.org/10.1186/s12913-016-1281-1>.

10. Crowe BL, Hailey DM and Carter R. Assessment of costs and benefits in the introduction of digital radiology systems. *Int J Biomed Comput* 1992; 30: 17-25. 1992/01/01. DOI: 10.1016/0020-7101(92)90058-z.

11. Doze S, Simpson J, Hailey D, et al. Evaluation of a telepsychiatry pilot project. *J Telemed Telecare* 1999; 5: 38-46.

12. Enam A, Torres-Bonilla J and Eriksson H. Evidence-based evaluation of ehealth interventions: Systematic literature review. *Journal of Medical Internet Research* 2018; 20. DOI: 10.2196/10971.

13. Goldstein D and Toth C. Thinking through e-health care return on investment. *Managed care interface* 2001; 14: 36-38.

14. Griscenko N. Comparison and evaluation of the Telehealth systems using a discrete event simulation. 2012.

15. Hailey D, Jacobs P, Simpson J, et al. An assessment framework for telemedicine applications. *J Telemed Telecare* 1999; 5: 162-170. 2000/01/11. DOI: 10.1258/1357633991933576.

16. Hailey DM and Crowe BL. Assessing the economic impact of telemedicine. *Disease Management and Health Outcomes* 2000; 7: 187-192. DOI: <http://dx.doi.org/10.2165/00115677-200007040-00002>.

17. Hailey D, Bulger T, Stayberg S, et al. The reality of applying an assessment guideline to a telemedicine mental health programme. *J Telemed Telecare* 2003; 9: 344-348; discussion 348-349.

18. Hailey D and Jennett P. The need for economic evaluation of telemedicine to evolve: the experience in Alberta, Canada. *Telemed J E Health* 2004; 10: 71-76.

19. Hakansson S and Gavelin C. What do we really know about the cost-effectiveness of telemedicine? *J Telemed Telecare* 2000; 6 Suppl 1: S133-136.

20. Hughes E, King C and Kitt S. Using the Australian and New Zealand Telehealth Committee framework to evaluate telehealth: identifying conceptual gaps. *J Telemed Telecare* 2002; 8 Suppl 3: S3:36-38.

21. Iribarren SJ, Cato K, Falzon L, et al. What is the economic evidence for mHealth? A systematic review of economic evaluations of mHealth solutions. *PLoS ONE* 2017; 12: e0170581. DOI: <https://dx.doi.org/10.1371/journal.pone.0170581>.

22. Jankovic D, Bojke L, Marshall D, et al. Systematic Review and Critique of Methods for Economic Evaluation of Digital Mental Health Interventions. *Appl Health Econ Health Policy* 2021; 19: 17-27. DOI: <https://dx.doi.org/10.1007/s40258-020-00607-3>.

23. Jean C, Duong TA, Stal-Le Cardinal J, et al. Sharing economic value between the stakeholders of a telehealth project: Methodological issues? *European Research in Telemedicine* 2016; 5: 37-44. DOI: <http://dx.doi.org/10.1016/j.eurtel.2016.04.002>.

24. Jurkeviciute M, van Velsen L, Eriksson H, et al. Identifying the Value of an eHealth Intervention Aimed at Cognitive Impairments: Observational Study in Different Contexts and Service Models. *Journal of Medical Internet Research* 2020; 22: e17720. DOI: <https://dx.doi.org/10.2196/17720>.

25. Kadu M, Ehrenberg N, Stein V, et al. Methodological quality of economic evaluations in integrated care: Evidence from a systematic review. *International Journal of Integrated Care* 2019; 19. DOI: 10.5334/ijic.4675.

26. Kennedy CA. The challenges of economic evaluations of remote technical health interventions. *Clin Invest Med* 2005; 28: 71-74.

27. Kidholm K, Bowes A, Dyrehauge S, et al. *The MAST Manual. MAST - Model for ASsessment of Telemedicine*. 2010.

28. Kidholm K, Ekeland AG, Jensen LK, et al. A model for assessment of telemedicine applications: MAST. *International Journal of Technology Assessment in Health Care* 2012; 28: 44-51. DOI: <https://dx.doi.org/10.1017/S0266462311000638>.

29. Kidholm K, Clemensen J, Caffery LJ, et al. The Model for Assessment of Telemedicine (MAST): A scoping review of empirical studies. *J Telemed Telecare* 2017; 23: 803-813. DOI: <https://dx.doi.org/10.1177/1357633X17721815>.

30. Kristiansen IS, Poulsen PB and Jensen KU. Economic aspects--saving billions with telemedicine: fact or fiction? *Curr* 2003; 32: 62-70.

31. Lobley D. The economics of telemedicine. *J Telemed Telecare* 1997; 3: 117-125.

32. Luxton DD. Considerations for planning and evaluating economic analyses of telemental health. *Psychol Serv* 2013; 10: 276-282. DOI: <https://dx.doi.org/10.1037/a0030658>.

33. Luzi D, Pecoraro F and Tamburis O. Economic evaluation of health IT. *Evidence-Based Health Informatics: Promoting Safety and Efficiency through Scientific Methods and Ethical Policy*. 2016, pp.165-180.

34. Mair FS, Haycox A, May C, et al. A review of telemedicine cost-effectiveness studies. *J Telemed Telecare* 2000; 6 Suppl 1: S38-40.

35. Ohinmaa A and Hailey D. Telemedicine, outcomes and policy decisions. *Disease Management and Health Outcomes* 2002; 10: 269-276. DOI: <http://dx.doi.org/10.2165/00115677-200210050-00001>.

36. Phillips KA, Douglas MP, Trosman JR, et al. "What Goes Around Comes Around": Lessons Learned from Economic Evaluations of Personalized Medicine Applied to Digital Medicine. *Value in Health* 2017; 20: 47-53. DOI: <https://dx.doi.org/10.1016/j.jval.2016.08.736>.

37. Powell A and Torous J. A Patient-Centered Framework for Measuring the Economic Value of the Clinical Benefits of Digital Health Apps: Theoretical Modeling. *JMIR Ment Health* 2020; 7: e18812. DOI: <https://dx.doi.org/10.2196/18812>.

38. Rojas SV and Gagnon MP. A systematic review of the key indicators for assessing telehomecare cost-effectiveness. *Telemed J E Health* 2008; 14: 896-904. DOI: <https://dx.doi.org/10.1089/tmj.2008.0009>.

39. Ruckdäschel S, Reiher M, Rohrbacher R, et al. The role of health economics in telemedicine. *Disease Management and Health Outcomes* 2006; 14: 3-7.

40. Rudolph SH and Levine SR. Telestroke, QALYs, and current health care policy: The Heisenberg uncertainty principle. *Neurology* 2011; 77: 1584-1585. DOI: <http://dx.doi.org/10.1212/WNL.0b013e31823433aa>.

41. Scott RE, McCarthy FG, Jennett PA, et al. Telehealth outcomes: a synthesis of the literature and recommendations for outcome indicators. *J Telemed Telecare* 2007; 13 Suppl 2: 1-38.

42. Sisk JE and Sanders JH. A proposed framework for economic evaluation of telemedicine. *Telemedicine Journal* 1998; 4: 31-37.

43. Suzuki T, Abe T, Tsuji S, et al. Survey on the willingness to pay for tele-health consultation. *Health Policy and Technology* 2019; 8: 248-253. DOI: <http://dx.doi.org/10.1016/j.hlpt.2019.07.004>.

44. Vis C, Bührmann L, Riper H, et al. Health technology assessment frameworks for eHealth: A systematic review. *International Journal of Technology Assessment in Health Care* 2020; 36: 204-216. DOI: 10.1017/S026646232000015X.

45. Wang F. Measuring adjusted quality of life in telemedicine. *Telemed J E Health* 2014; 20: 338-341. DOI: <https://dx.doi.org/10.1089/tmj.2013.0159>.

46. Zanaboni P and Lettieri E. Institutionalizing telemedicine applications: The challenge of legitimizing decision-making. *Journal of Medical Internet Research* 2011; 13. DOI: 10.2196/jmir.1669.

47. Angjellari-Dajci F, Lawless WF, Stachura ME, et al. Economic evaluations for service delivery in autism spectrum disorders: Benefit-cost analysis for emerging telehealth systems. *Handbook of Research on ICTs and Management Systems for Improving Efficiency in Healthcare and Social Care*. 2013, pp.16-42.

48. Davalos ME, French MT, Burdick AE, et al. Economic evaluation of telemedicine: review of the literature and research guidelines for benefit-cost analysis. *Telemed J E Health* 2009; 15: 933-948. DOI: <https://dx.doi.org/10.1089/tmj.2009.0067>.

49. Le Goff-Pronost M and Sicotte C. The added value of thorough economic evaluation of telemedicine networks. *European Journal of Health Economics* 2010; 11: 45-55. DOI: <https://dx.doi.org/10.1007/s10198-009-0162-5>.

50. Bergmo TS. How to Measure Costs and Benefits of eHealth Interventions: An Overview of Methods and Frameworks. *Journal of Medical Internet Research* 2015; 17: e254. DOI: <https://dx.doi.org/10.2196/jmir.4521>.

51. National Institute for Health and Care Excellence. *Evidence standards framework for digital health technologies*. 2018. National Institute for Health and Care Excellence London, UK.

52. Bongiovanni-Delaroziere I and Le Goff-Pronost M. Economic evaluation methods applied to telemedicine: From a literature review to a standardized framework. *European Research in Telemedicine* 2017; 6: 117-135. DOI: <http://dx.doi.org/10.1016/j.eurtel.2017.08.002>.

53. Snoswell CL, Caffery LJ, Haydon HM, et al. A cost-consequence analysis comparing patient travel, outreach, and telehealth clinic models for a specialist diabetes service to Indigenous people in Queensland. *Journal of Telemedicine and Telecare* 2019; 25: 537-544. DOI: 10.1177/1357633X19873239.

54. Noble SM, Coast J and Benqer JR. A Cost-Consequences Analysis of Minor Injuries Telemedicine. *Journal of Telemedicine and Telecare* 2005; 11: 15-19. DOI: 10.1177/1357633X0501100104.

55. Langabeer JR, 2nd, Champagne-Langabeer T, Alqusairi D, et al. Cost-benefit analysis of telehealth in pre-hospital care. *J Telemed Telecare* 2017; 23: 747-751. 2016/12/04. DOI: 10.1177/1357633x16680541.

56. Stahl JE and Dixon RF. Acceptability and willingness to pay for primary care videoconferencing: a randomized controlled trial. *J Telemed Telecare* 2010; 16: 147-151. 2010/04/14. DOI: 10.1258/jtt.2009.090502.

57. Chang J, Savage SJ and Waldman DM. Estimating Willingness to Pay for Online Health Services with Discrete-Choice Experiments. *Appl Health Econ Health Policy* 2017; 15: 491-500. 2017/03/16. DOI: 10.1007/s40258-017-0316-z.

58. Noben C, Evers S, Genabeek JV, et al. Improving a web-based employability intervention for work-disabled employees: results of a pilot economic evaluation. *Disabil Rehabil Assist Technol* 2017; 12: 280-289. 2016/01/26. DOI: 10.3109/17483107.2015.1135999.

59. Tan E, Gao L, Tran HNQ, et al. Telestroke for acute ischaemic stroke: A systematic review of economic evaluations and a de novo cost–utility analysis for a middle income country. *Journal of Telemedicine and Telecare* 2021: 1357633X211032407. DOI: 10.1177/1357633X211032407.

60. Naveršnik K and Mrhar A. Routine Real-Time Cost-Effectiveness Monitoring of a Web-Based Depression Intervention: A Risk-Sharing Proposal. *J Med Internet Res* 2014; 16: e67. Viewpoint 27.02.2014. DOI: 10.2196/jmir.2592.

61. Dakin H, Abel L, Burns R, et al. Review and critical appraisal of studies mapping from quality of life or clinical measures to EQ-5D: an online database and application of the MAPS statement. *Health Qual Life Outcomes* 2018; 16: 31. 2018/02/13. DOI: 10.1186/s12955-018-0857-3.

62. Franzini L, Sail KR, Thomas EJ, et al. Costs and cost-effectiveness of a telemedicine intensive care unit program in 6 intensive care units in a large health care system. *J Crit Care* 2011; 26: 329.e321-326. 2011/03/08. DOI: 10.1016/j.jcrc.2010.12.004.

63. LeFevre AE, Shillcutt SD, Broomhead S, et al. Defining a staged-based process for economic and financial evaluations of mHealth programs. *Cost Eff Resour Alloc* 2017; 15: 5. DOI: <https://dx.doi.org/10.1186/s12962-017-0067-6>.

64. Fottrell E, Ahmed N, Morrison J, et al. Community groups or mobile phone messaging to prevent and control type 2 diabetes and intermediate hyperglycaemia in Bangladesh (DMagic): a cluster-randomised controlled trial. *Lancet Diabetes Endocrinol* 2019; 7: 200-212. DOI: 10.1016/S2213-8587(19)30001-4.

65. Haghparast-Bidgoli H, Shaha SK, Kuddus A, et al. Protocol of economic evaluation and equity impact analysis of mHealth and community groups for prevention and control of diabetes in rural Bangladesh in a three-arm cluster randomised controlled trial. *BMJ Open* 2018; 8: e022035. 2018/08/22. DOI: 10.1136/bmjopen-2018-022035.

66. Pires M, Shaha S, King C, et al. Equity impact of participatory learning and action community mobilisation and mHealth interventions to prevent and control type 2 diabetes and intermediate hyperglycaemia in rural Bangladesh: analysis of a cluster randomised controlled trial. *Journal of Epidemiology and Community Health* 2022: jech-2021-217293. DOI: 10.1136/jech-2021-217293.

67. Witt Udsen F, Lilholt PH, Hejlesen OK, et al. Subgroup analysis of telehealthcare for patients with chronic obstructive pulmonary disease: the cluster-randomized Danish Telecare North Trial. *Clinicoecon Outcomes Res* 2017; 9: 391-401. DOI: 10.2147/CEOR.S139064.

68. Khairat S, Haithcoat T, Liu S, et al. Advancing health equity and access using telemedicine: a geospatial assessment. *J Am Med Inform Assoc* 2019; 26: 796-805. 2019/07/25. DOI: 10.1093/jamia/ocz108.
